# Periodontitis salivary microbiota exacerbates colitis-induced anxiety-like behavior via gut microbiota

**DOI:** 10.1038/s41522-023-00462-9

**Published:** 2023-12-07

**Authors:** Jun Qian, Jiangyue Lu, Shuyu Cheng, Xihong Zou, Qing Tao, Min Wang, Nannan Wang, Lichun Zheng, Wenzheng Liao, Yanfen Li, Fuhua Yan

**Affiliations:** 1 Nanjing Stomatological Hospital, Affiliated Hospital of Medical School, Nanjing University, Nanjing, China; 2 Center for Translational Medicine and Jiangsu Key Laboratory of Molecular Medicine, Medical School, Nanjing University, Nanjing, China

**Keywords:** Dentistry, Microbiology, Microbiota

## Abstract

The gut–brain axis is a bidirectional communication system between the gut and central nervous system. Many host-related factors can affect gut microbiota, including oral bacteria, making the brain a vulnerable target via the gut–brain axis. Saliva contains a large number of oral bacteria, and periodontitis, a common oral disease, can change the composition of salivary microbiota. However, the role and mechanism of periodontitis salivary microbiota (PSM) on the gut–brain axis remain unclear. Herein, we investigated the nature and mechanisms of this relationship using the mice with dextran sulfate sodium salt (DSS)-induced anxiety-like behavior. Compared with healthy salivary microbiota, PSM worsened anxiety-like behavior; it significantly reduced the number of normal neurons and activated microglia in DSS mice. Antibiotic treatment eliminated the effect of PSM on anxiety-like behavior, and transplantation of fecal microbiota from PSM-gavaged mice exacerbated anxiety-like behavior. These observations indicated that the anxiety-exacerbating effect of PSM was dependent on the gut microbiota. Moreover, the PSM effect on anxiety-like behavior was not present in non-DSS mice, indicating that DSS treatment was a prerequisite for PSM to exacerbate anxiety. Mechanistically, PSM altered the histidine metabolism in both gut and brain metabolomics. Supplementation of histidine-related metabolites had a similar anxiety-exacerbating effect as that of PSM, suggesting that histidine metabolism may be a critical pathway in this process. Our results demonstrate that PSM can exacerbate colitis-induced anxiety-like behavior by directly affecting the host gut microbiota, emphasizing the importance of oral diseases in the gut–brain axis.

## Introduction

Both the gut and brain are receptor organs affected by external factors that trigger responses reflecting the body’s physiological state; their interaction is known as the gut–brain axis. The cross-talk of inflammation along the gut–brain axis affects immune, neuronal, and other cells, playing a key role in regulating physiological behavior and inflammatory responses^1^. Moreover, emerging evidence indicates that gut microbiota can regulate gut-derived metabolites that are dispersed in various organs, including the brain^2,3^. Gut microbiota can also modulate neurotransmitter synthesis, such as serotonin and dopamine, which act on target cells in the gut and affect the activity of the nervous system activity through the vagus nerve^4,5^. Therefore, it is necessary to explore the mechanisms underlying the signals of the gut–brain axis under physiological and pathological conditions.

Oral diseases can affect gut microbiota through saliva, which contains a large number of oral bacteria. Periodontitis is a chronic non-communicable disease caused by dysbiosis of the subgingival microbiota and characterized by gingival inflammation, periodontal attachment loss and alveolar bone resorption^6–9^. It has been reported to be strongly associated with several diseases, including inflammatory bowel disease (IBD)^10^, and Alzheimer’s disease (AD)^7,11^. The salivary microbiota of patients with periodontitis differs markedly from that of healthy individuals; their representative bacteria are *Porphyromonas gingivalis* (*P. gingivalis*) and *Fusobacterium* genus^12^. Remarkably, a substantial number of oral bacteria are swallowed into the gastrointestinal tract daily (a person swallows approximately 10^12^–10^13^ bacteria)^13,14^. Despite the presence of gastric acid, some of these oral bacteria can partially survive gastric acid exposure and disrupt the intestinal function and immune homeostasis^15,16^ *P. gingivalis* has been detected in the ileum at 3 h and in the colon at 16 h after oral ingestion, inducing dysbiosis in gut microbiota^14^. Thus, periodontitis can affect the gut microbiota through the saliva. Previous studies have also confirmed that both periodontitis and salivary microbiota from patients with periodontitis can alter the composition of gut microbiota^17–19^. However, the exact role and mechanism of periodontitis salivary microbiota on the gut–brain axis remains to be elucidated.

Anxiety is a common psychiatric disorder that is characterized by symptoms such as social fear, panic, and avoidance behavior, which negatively affects the quality of life^20,21^. Anxiety is related to central nervous system abnormalities, such as activation of microglia in the brain and destruction of nerves^22^. It is also strongly associated with intestinal lesions and gut microbiota disorders. Up to one-third of patients with IBD experience anxiety-like reactions^23^. Transplantation of fecal microbiota from patients with IBD exacerbated anxiety-like reactions in mice and, conversely, transplantation of that from healthy individuals improved anxiety-like reactions^24,25^. Therefore, we believe that mice treated with dextran sulfate sodium (DSS), which causes both colitis and anxiety, are an appropriate animal behavioral model to explore the gut–brain axis. Periodontitis affects colitis and gut microbiota via the oral–gut axis^26^. Our previous study also showed that periodontitis salivary microbiota exacerbated colitis and disrupted the intestinal barrier^17^. Additionally, periodontitis can affect neuropsychiatric behaviors. A systematic review demonstrated that patients with periodontitis exhibit higher anxiety^27^. Previous studies have also shown that oral bacteria can be found in the brain, and that periodontitis-related bacteria affects the development of AD by increasing neuroinflammation and damaging Tau proteins in the brain^28,29^. Based on these observations, we hypothesized that periodontitis-associated microbiota can aggravate DSS-induced anxiety-like behaviors through gut microbiota disorders.

To test this hypothesis, we collected salivary microbiota from healthy individuals and patients with periodontitis and gavaged them into DSS-induced mice to observe changes in anxiety-like behavior and brain lesions. We then verified the key role of gut microbiota in the effect of periodontitis salivary microbiota on anxiety-like behavior. Finally, we investigated the possible mechanisms related to gut microbiota and metabolites. We aimed to reveal the role of periodontitis in the gut–brain axis using this model, emphasizing the importance of preventing periodontal disease in patients with mental disorders.

## Results

### Periodontitis salivary microbiota exacerbated anxiety-like behaviors in DSS mice

**Fig. 1:**
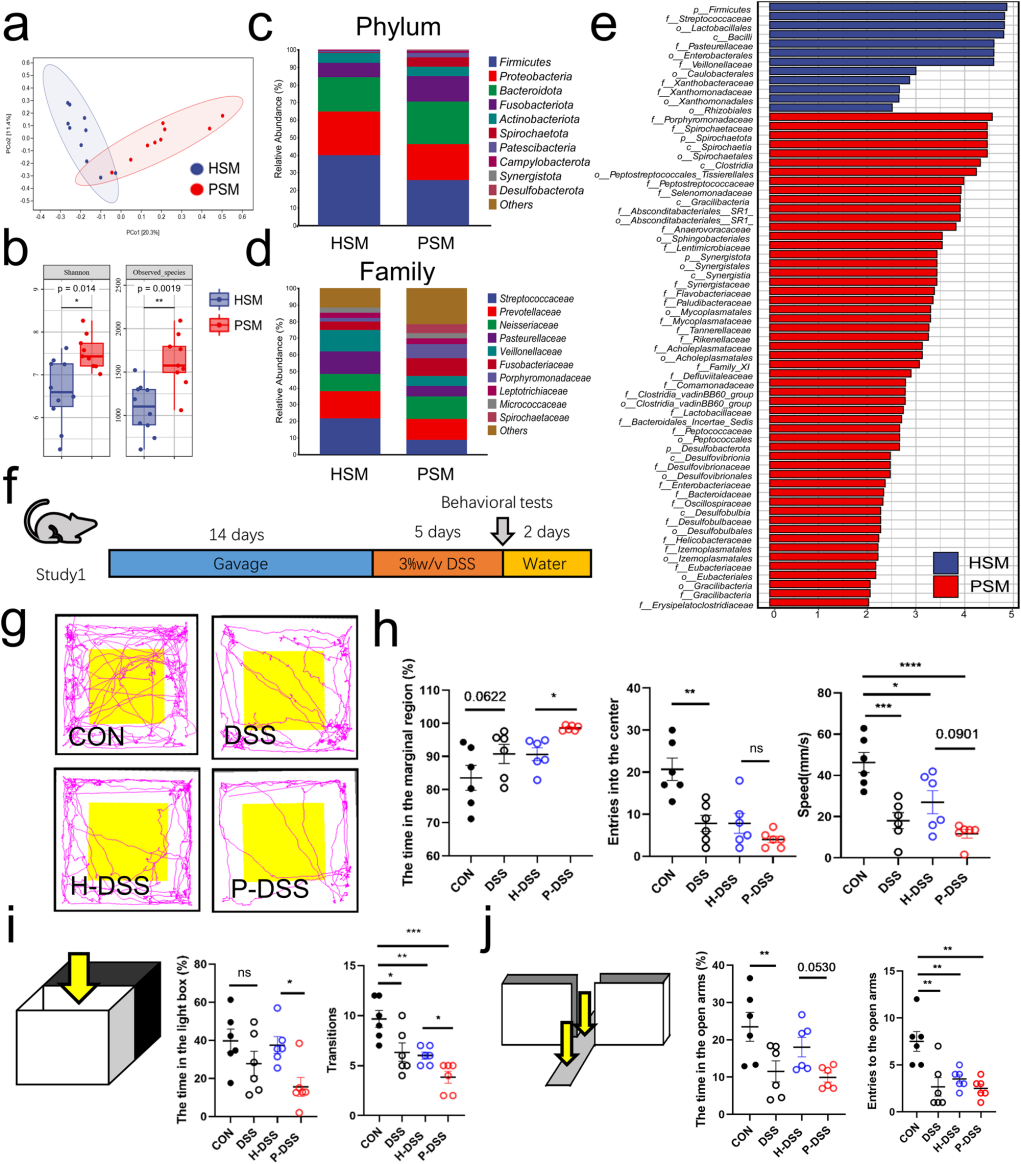
Periodontitis salivary microbiota exacerbate anxiety-like behavior in dextran sulphate sodium (DSS) mice. **a** Principal coordination analysis of the healthy salivary microbiota (HSM) and periodontitis salivary microbiota (PSM) (Bray-Curtis; HSM:*n* = 10, PSM:*n* = 9). **b** α-diversity (Shannon, and observed species) of HSM and PSM; each box plot represents the median, interquartile range, minimum, and maximum values (Wilcoxon). **c** Composition of the gut microbiota at the phylum level in HSM and PSM. **d** Composition of the gut microbiota at the family level in HSM and PSM groups. **e** The discriminative biomarkers in the group of HSM (bule) and PSM (red) according to the LEfSe analysis. **f** Schematic representation and study design. A detailed description is provided in study 1 of the Methods section (*n* = 6 per group). **g** Representative image of the open-field track diagram. **h** Time spent in the marginal region, speed in the marginal region, and frequency of entering the centre zone in the open field. **i** Time spent in the light box and the frequency of light-dark transitions. **j** Time spent in the open arm and the number of times the subjects entered the open arms in the elevated plus-maze. Statistical analysis was performed by the ordinary one-way ANOVA test with Tukey’s correction. Results are shown as mean ± standard error of mean. *p < 0.05, **p < 0.01, ***p < 0.001.

Salivary microbiota was collected from a total of 19 individuals, including 9 patients with periodontitis and 10 healthy individuals. Principal coordinate analysis (PCoA) based on Bray–Curtis revealed differences between periodontitis salivary microbiota (PSM) and healthy salivary microbiota (HSM) (Fig. 1a). The α-diversity of PSM was significantly higher than that of HSM (Fig. 1b). At the phylum level, the relative abundance of *Firmicutes* was decreased in the PSM, compared with that in the HSM (Fig. 1c). At the family level, the relative abundance of *Streptococcaceae* was decreased, and that of *Porphyromonadaceae* was increased in the PSM, compared with those in the HSM (Fig. 1d). According to linear discriminant analysis effect size (LEfSe) analysis (LDA > 2), *Porphyromonadaceae* and *Spirochaetaceae* were the main representative family-level taxa in the PSM, while *Streptococcaceae* was the representative taxa in the HSM at the family level (Fig. 1e). Analysis of the composition of microbiomes (ANCOM) revealed that, *Spirochaetaceae* and *Synergistaceae*, which were upregulated in the PSM, and *Streptococcaceae*, which was upregulated in the HSM, were the most distinctive taxa present between PSM and HSM (Supplementary Fig. 1a). These results showed that PSM differed significantly from HSM, with *Porphyromonadaceae* and *Spirochaetaceae* as the main characteristic bacteria in the PSM and *Streptococcaceae* as the main characteristic bacteria in the HSM (Supplementary Fig. 1b). We gavaged the mixed salivary microbiota into a total of 24 mice and successfully induced colitis using DSS (Fig. 1f). Consistent with our previous observation, compared with HSM (H-DSS group), PSM (P-DSS group) exacerbated the colitis of DSS-induced mice, including decreasing the colonic length, increasing histological scores, and disrupting intestinal barriers (Supplementary Fig. 1c–g).

To observe the effect of salivary microbiota on anxiety-like behavior, behavioral tests based on open field, light–dark transitions and elevated plus-maze were conducted. In the open field, the DSS group spent more time in the marginal region, fewer times in the center zone, and was slower than the CON group. Time spent in the marginal region was higher and speed was lower in the P-DSS group than in the H-DSS group (Fig. 1g, h). In light–dark transitions, the frequency of transitions in the DSS group was significantly lower than that in the CON group. Time spent in the light box and the frequency of transitions between the light and dark boxes were significantly lower in the P-DSS group compared with those in the H-DSS group (Fig. 1i). In the elevated plus-maze test, the open-arm activity time and number of times the mice entering the open arms were lower in the DSS group than they were in the CON group, and the open-arm activity time was also reduced in the P-DSS group compared with that in the H-DSS group (Fig. 1j). The above results show that DSS exacerbates anxiety-like behavior, while PSM exacerbated DSS-induced anxiety-like behavior. However, before DSS treatment, the behavioral changes were not significantly different between the PSM-gavaged and HSM-gavaged mice, suggesting that DSS treatment is an important prerequisite for this exacerbation (Supplementary Fig. 2a–d).

### Gut microbiota mediated the effect of PSM exacerbating DSS-induced anxiety-like behavior

**Fig. 2:**
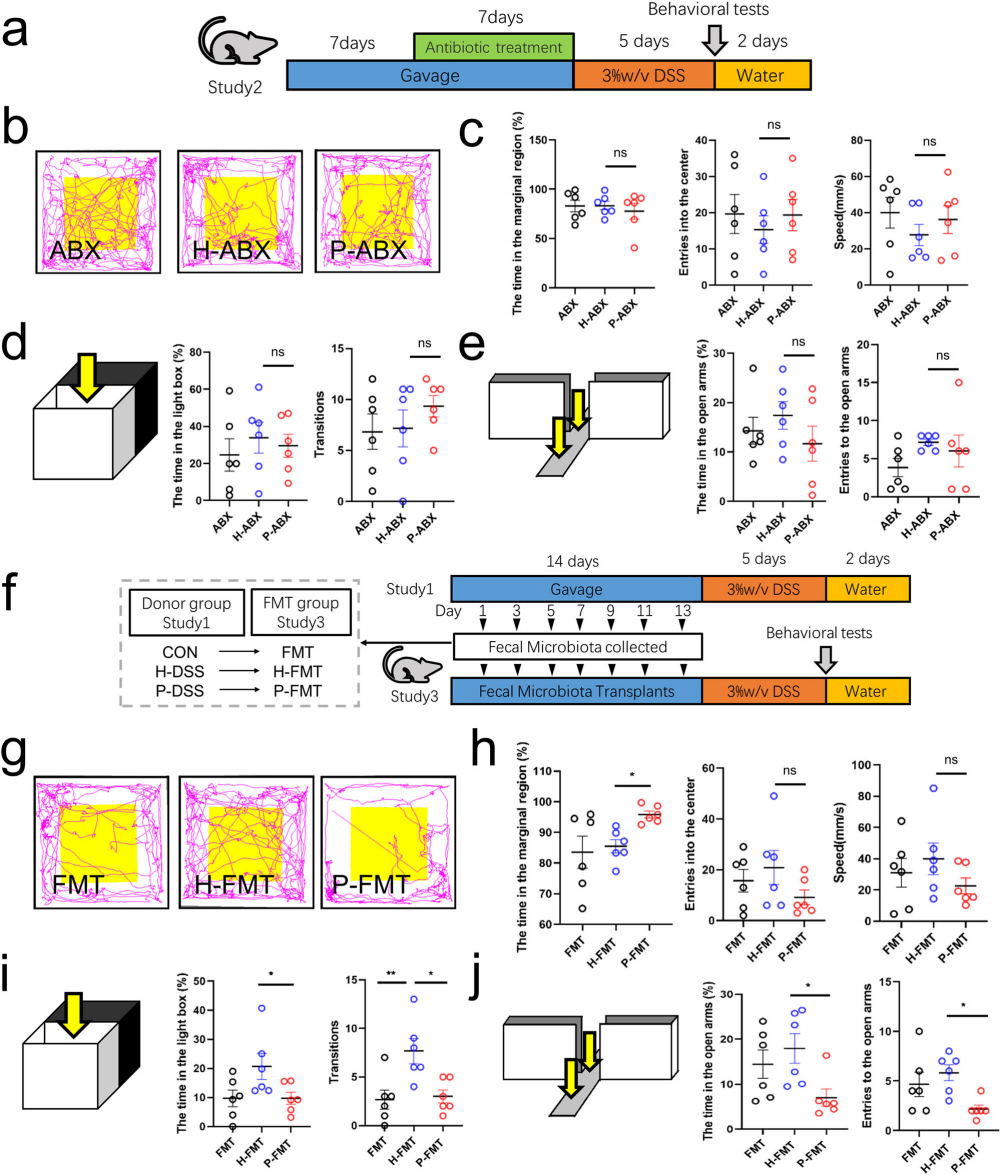
Gut microbiota is key in periodontitis, and salivary microbiota exacerbate dextran sulphate sodium-induced anxiety-like behavior. **a** Study design for study 2. A detailed description is provided in the Methods section (*n* = 6 per group). **b** Representative image of the open-field track diagram after antibiotic treatment. **c** Time spent in the marginal region, speed in the marginal region, and number of times entering the centre zone in the open field. **d** Time spent in the light box and frequency of light-dark transitions. **e** Time spent in the open arm and the number of times the mice entered the open arms in the elevated plus-maze. **f** Schematic representation and study design of faecal microbiota transplantation. The faecal microbiota was from study 1. The details are presented in the Methods section (*n* = 6 per group). **g** Representative open-field track diagram of the faecal microbiota transplantation. **h** Time spent in the marginal region, the frequency of the mice entered the centre zone, and speed in the marginal region in the open field. **i** Time spent in the light box and frequency of light-dark transitions. **j** Time in the open arm and the number of times the mice entered the open arms in the elevated plus-maze. Statistical analysis was performed by the ordinary one-way ANOVA test with Tukey’s correction. Results are shown as mean ± standard error of mean. *p < 0.05, **p < 0.01.

Previous studies have shown that PSM causes gut microbiota disorders and that gut microbiota can influence neuropsychiatric diseases^19^; therefore, we hypothesized that gut microbiota affected by PSM plays a key role in the exacerbation of DSS-induced anxiety-like behavior. To investigate the effect of gut microbiota altered by salivary microbiota on anxiety-like behavior, we negated the differences in antibiotic-sensitive gut microbiota between the groups using antibiotics treatment. We treated DSS mice with antibiotics for 1 week after salivary microbiota gavage (Fig. 2a). No significant difference was noted in activity time or speed in the marginal region or in times entering the center zone in the open field between the ABX, H-ABX, and P-ABX groups (Fig. 2b, c). The results of light–dark transitions and elevated plus-maze were also not significantly different between the groups after antibiotic treatment (Fig. 2d, e). These results indicated that the antibiotic treatment eliminated the behavioral differences between the two groups, suggesting that gut microbiota is a key factor in the PSM exacerbating anxiety-like behaviors.

To further verify the role of gut microbiota in anxiety-like behaviors, we transplanted fresh fecal microbiota from the salivary microbiota-gavaged mice and performed DSS treatment to observe behavioral changes (Fig. 2f). DSS mice spent significantly more time in the marginal area of the open field in the P-FMT group than those in the H-FMT group. They also showed a reduced speed in the marginal region and frequency of entering the center zone in the open field, but not significantly (Fig. 2g, h). Time spent in the light, the frequency of transitions between light and dark boxes, open-arm activity time, and times entering the open arms were significantly decreased (Fig. 2i, j) in the P-FMT group compared with those in the H-FMT group. Notably, the frequency of transitions between light and dark boxes was significantly increased in the H-DSS group compared with that in the P-FMT and FMT groups (Fig. 2i). These behavioral results showed that the P-FMT group exerted an exacerbating anxiety-like effect similar to that of PSM, confirming that PSM exacerbates DSS-induced anxiety-like behavior via the gut microbiota. In addition, we observed anxiety-like behavior in the non-DSS mice. Before DSS treatment, no significant differences in open field, light–dark transitions, or elevated plus-maze were identified between the H-ABX, and P-ABX groups (Supplementary Fig. 3a–e), or between the H-FMT and P-FMT groups (Supplementary Fig. 3f–j), suggesting that DSS treatment is a prerequisite for the effect of PSM on anxiety.

### PSM impaired neurons and activated microglia in the cerebral cortex via the gut microbiota

**Fig. 3:**
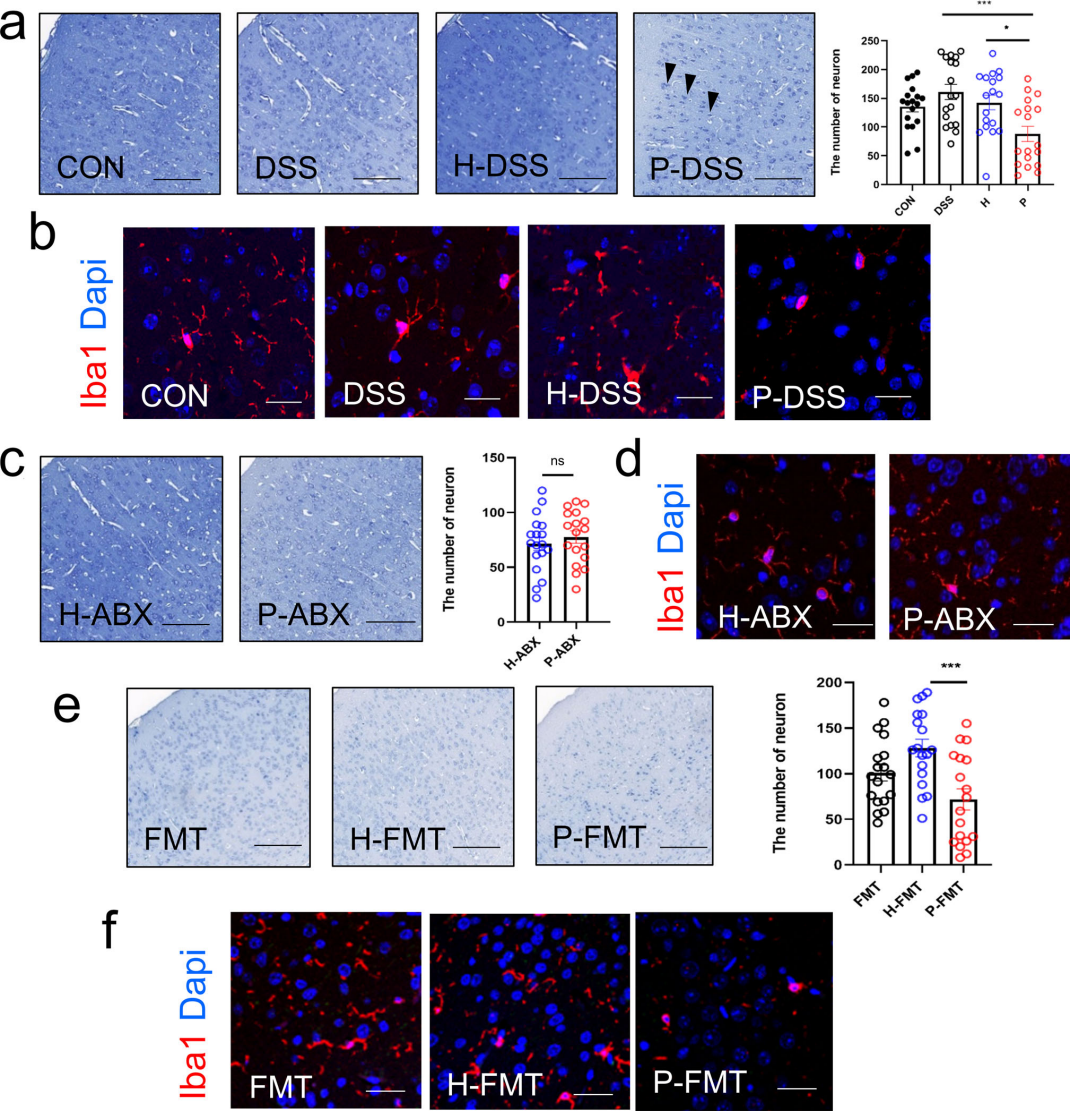
Periodontitis salivary microbiota reduce neuronal numbers and activate cerebral cortex microglia via gut microbiota. **a** Representative image of Nissl staining and the number of neurons in the CON, DSS, H-DSS, and P-DSS groups, scale bar = 150 μm. **b** Representative image of iba1 (red) and DAPI (blue) staining of microglial cells in the CON, DSS, H-DSS, and P-DSS groups, scale bar = 50 μm. **c** Representative image of Nissl staining and number of neurons after antibiotic treatment in the H-ABX and P-ABX groups, scale bar = 150 μm. **d** Representative image of iba1 (red) and DAPI (blue) staining of microglial cells in the H-ABX and P-ABX groups, scale bar = 50 μm. **e** Representative image of Nissl staining and the number of neurons after faecal microbiota transplantation, scale bar = 150 μm. **f** Representative image of iba1 (red) and DAPI (blue) staining of microglial cells in the faecal microbiota transplantation experiment, scale bar = 50 μm. Statistical analysis was performed by the one-way ANOVA test with Tukey’s correction (**a**, **e**) and two-tailed *t* test (**c**). Results are shown as mean ± standard error of mean. *p < 0.05, ***p < 0.001. *n* = 6 per group; For normal neurons counting,6 mice per group, and 3 views of the same site were selected for each mouse to be counted.

Next, we explored the anxiety-related pathological changes in the process of PSM affecting anxiety-like behavior, including the morphology of microglia by immunofluorescence and the number of normal neurons by Nissl staining^22^. According to the results of Nissl staining, compared with the H-DSS group, the number of normal neurons was significantly decreased in the cerebral cortex in the P-DSS group, characterized by cytoplasmic consolidation, irregular shape and deepening of staining in neurons (black arrows; Fig. 3a). Staining for ionized calcium‑binding adapter molecule 1 (Iba1), a biomarker of microglia, revealed significant microglial cell crinkling in the P-DSS group compared with that in the H-DSS group in the cerebral cortex, indicating that microglia were activated (Fig. 3b). However, no significant changes were observed in the morphology of neurons in the hippocampal dentate gyrus (DG) and CA regions among the CON, DSS, H-DSS, and P-DSS groups (Supplementary Fig. 4a, b). There also were no significant differences in microglial alterations in the hippocampal DG and CA regions between the groups (Supplementary Fig. 5a, b). The above findings showed that PSM altered the brain pathology in the cerebral cortex.

To determine the role of gut microbiota is the alteration of brain pathology, we also detected microglia and neurons in antibiotic treatment and fecal microbiota transplantation mice. In the antibiotic treatment mice, all the differences in the number of neurons and microglial cell activation disappeared between the H-ABX and P-ABX groups (Fig. 3c, d). Similar to the effect of PSM, the results of the fecal microbiota transplantation experiment showed a significant decrease in the number of neurons and activation of microglia in the cerebral cortex in the P-FMT group compared with that in the H-FMT group (Fig. 3e, f). Thus, PSM can reduce the number of normal neurons and activate microglia via the gut microbiota, which can exacerbate anxiety-like behavior.

### PSM altered the composition of gut microbiota in DSS mice

**Fig. 4:**
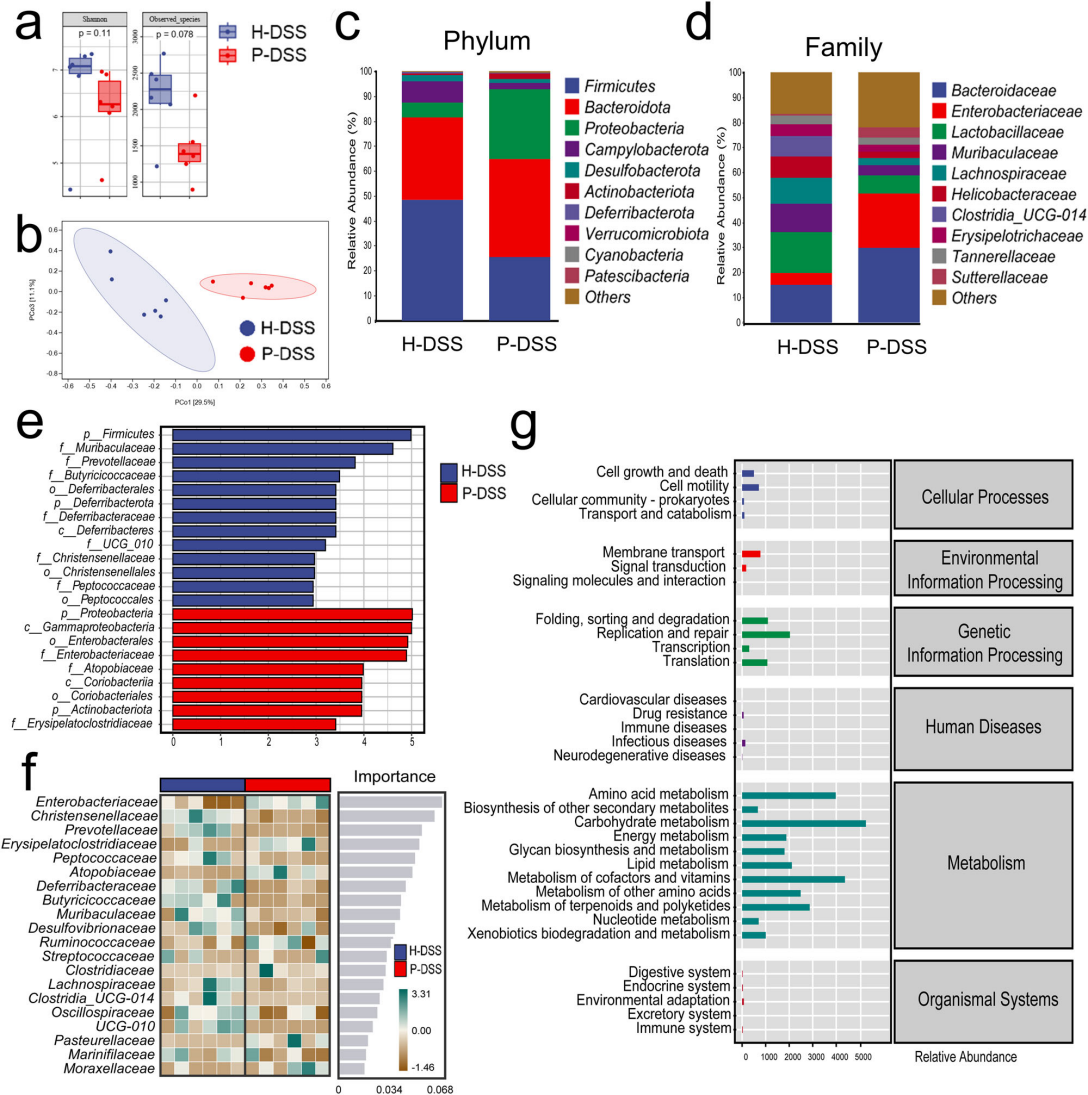
Periodontitis salivary microbiota alter the composition of gut microbiota. **a** α-Diversity (Shannon, observed species) of bacterial species in the H-DSS and P-DSSgroups; each box plot represents the median, interquartile range, minimum, and maximum values (Wilcoxon; *n* = 6 per group). **b** Principal coordination analysis of the H-DSS and P-DSS groups (Bray-Curtis). **c** Composition of the gut microbiota at the phylum level in the H-DSS and P-DSS groups. **d** Composition of the gut microbiota at the family level in the H-DSS and P-DSS groups. **e** The discriminative biomarkers in the group of H-DSS (bule) and P-DSS (red) groups indicated by LEfSe analysis. **f** Important bacteria based on the random forest analysis. **g** Functional prediction of gut microbiota according to PICRUSt2 in the H-DSS and P-DSS groups.

To investigate the alterations in the gut microbiota, we examined gut microbiota composition in the H-DSS and P-DSS groups using 16 S gene sequencing. Compared with the H-DSS group, the P-DSS group showed a decrease in α-diversity (Fig. 4a). PCoA revealed significant differences in gut microbiota composition between the two groups (Fig. 4b). At the phylum level, the relative abundance of *Firmicutes* was decreased in the P-DSS group than that in the H-DSS group (Fig. 4c). At the family level, the relative abundance of *Enterobacteriaceae* and *Bacteroidaceae* were increased in the P-DSS group than those in the H-DSS group (Fig. 4d). LEfSe analysis indicated that the representative microbiota of the P-DSS group belonged to the *Enterobacteriaceae* family, whereas the H-DSS group was represented by the *Firmicutes* phylum, the *Muribaculacae* and *Prevotellaceae* families (Fig. 4e). The results of the random forest analysis predicted that *Enterobacteriaceae* and *Christensenellaceae* might be important bacterial groups that play a role in the P-DSS group (Fig. 4f). The results of ANCOM revealed that *Atopobiaceae, Enterobacteriaceae* and *Erysipelatoclostridiaceae*, which were upregulated in the P-DSS group, and *Butyricicoccaceae*, which was upregulated in the H-DSS group, were the most distinctive taxa between the P-DSS and H-DSS groups (Supplementary Fig. 6a). Thus, *Enterobacteriaceae* might be the key pathogenic bacteria in the P-DSS group. In addition, *Atopobiaceae, Muribaculacae* and *Bacteroidaceae* differed significantly between the H-DSS and P-DSS groups (Supplementary Fig. 6b). *Klebsiella spp*. and *Enterobacter spp*. were the main species of *Enterobacteriaceae*; thus, we examined their expression using PCR and found that *Klebsiella spp*. was significantly upregulated in the P-DSS group (Supplementary Fig. 6c). Finally, the functional prediction of the gut microbiota of the H-DSS and P-DSS groups based on Phylogenetic Investigation of Communities by Reconstruction of Unobserved States (PICRUSt2)-tagged gene sequences showed that the main functional pathways of these bacteria are enriched in metabolism-related tasks, including amino acid metabolism, carbohydrate metabolism, and metabolism of cofactors and vitamins (Fig. 4g). Genetic information processing is mainly enriched in replication and repair, whereas cellular processes are mainly enriched in cell motility. These findings suggested that PSM led to gut microbiota disorder, and that these disturbances may be related to alterations in metabolites polarized to amino acid and carbohydrate metabolism.

### Alteration of gut metabolites caused by PSM in DSS mice

**Fig. 5:**
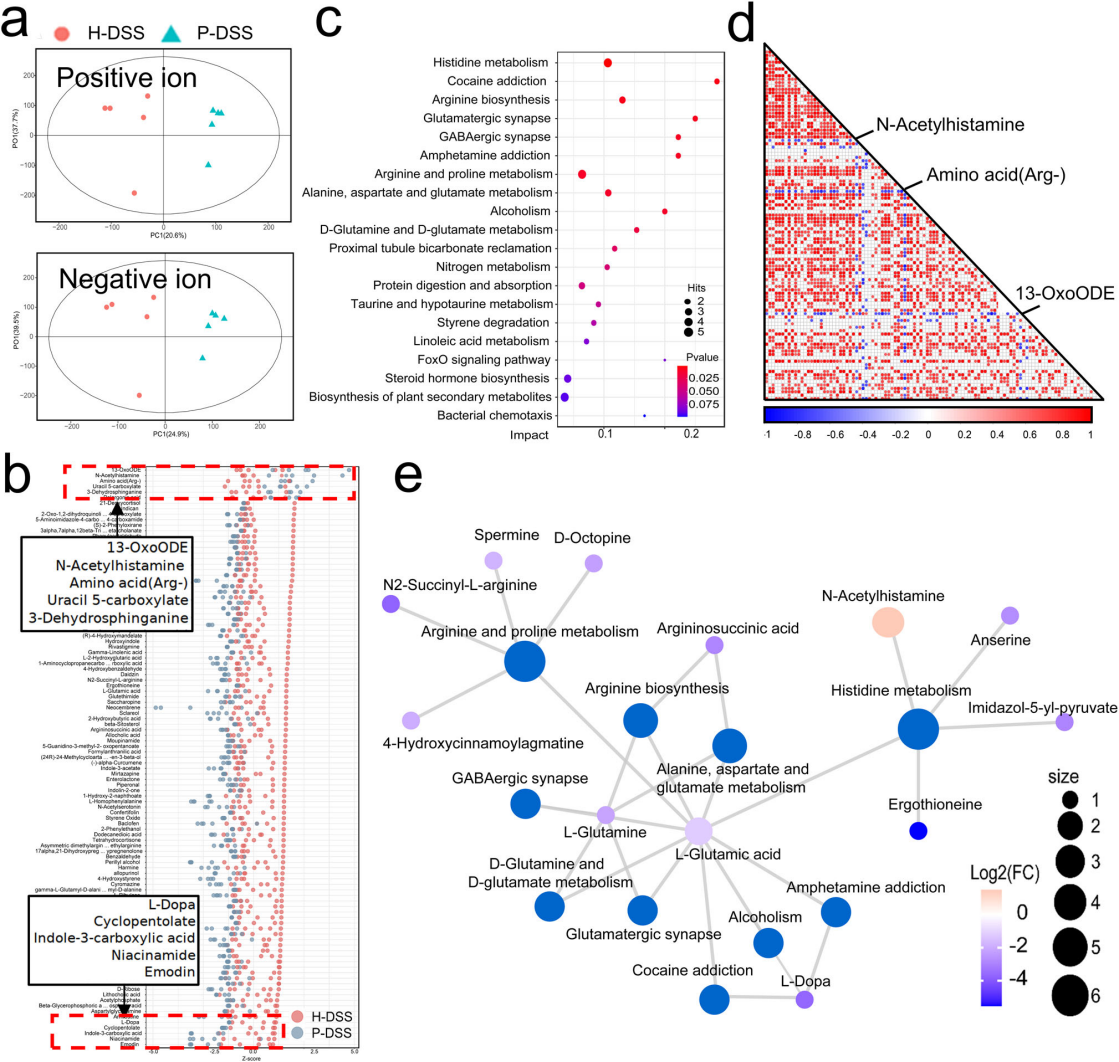
Alteration of gut metabolites after gavage with periodontitis salivary microbiota. **a** OPLS-DA score plots of positive and negative ion modes (*n* = 5 per group). **b** Z-score analysis showing metabolite expression in the P-DSS and H-DSS groups. **c** Spearman correlation coefficient analysis of the different metabolites in the P-DSS and H-DSS groups. **d** Enrichment analysis of the gut metabolites in the P-DSS and H-DSS groups. The impact of the pathway shown on the horizontal axis. **e** Network for different metabolites and their pathways. Each blue plot represents a pathway and the size of the blue plot means the number of metabolites contained in the pathway. The other plot represents the metabolite and the colour of the other plot represents the log2(FC) of the P-DSS group to the H-DSS group.

We explored gut metabolite changes using liquid chromatography-mass spectrometry. Orthogonal partial least-squares discriminant (OPLS-DA) analysis of metabolites screened in positive and negative ion modes was performed separately, which revealed significant differences in intestinal metabolites between the P-DSS and H-DSS groups (Fig. 5a). Z-score analysis was performed to observe the relative levels of these metabolites; the top five most abundant metabolites in the P-DSS group were 13-OxoCOE, N-acetylhistamine (NAH), amino acid (arg-), uracil 5-carboxylate, and 3-dehydrosphinganine, while emodin, niacinamide, indole-3-carboxylic acid, cyclopentolate, and L-dopa were the top five most abundant metabolites in the H-DSS group (Fig. 5b). Enrichment analyses of the differential metabolites revealed that amino acid metabolism (histidine metabolism and arginine biosynthesis) and neurotransmitter-related pathways (cocaine addiction, glutamatergic synapses, and GABAergic synapses) were the main pathways, and of these, histidine metabolism markedly varied (Fig. 5c). Spearman correlation analysis was used to identify the correlation between the metabolites, and NAH and amino acid (arg-) had a significantly opposite trend compared with the other metabolites, suggesting a possible key role of these metabolites (Fig. 5d). Finally, a network of these metabolites and their pathways was constructed. According to this network, amino acid metabolism (such as histidine, arginine, and proline) comprised the pathways in which most metabolites were involved, with L-glutamic acid and L-glutamine being the key nodes, and NAH was the only metabolite involved in these pathways that was highly expressed in the P-DSS group (Fig. 5e). Notably, some gut metabolites and related pathways, such as GABAergic synapses and L-dopa (high in the H-DSS group), are neurotransmitters involved in anxiety-like behavior. Therefore, we believed that these metabolites, especially histidine metabolism and NAH, were key to the worsening of anxiety-like behaviors due to PSM.

### Abnormalities in brain metabolism caused by PSM in DSS-treated mice

**Fig. 6:**
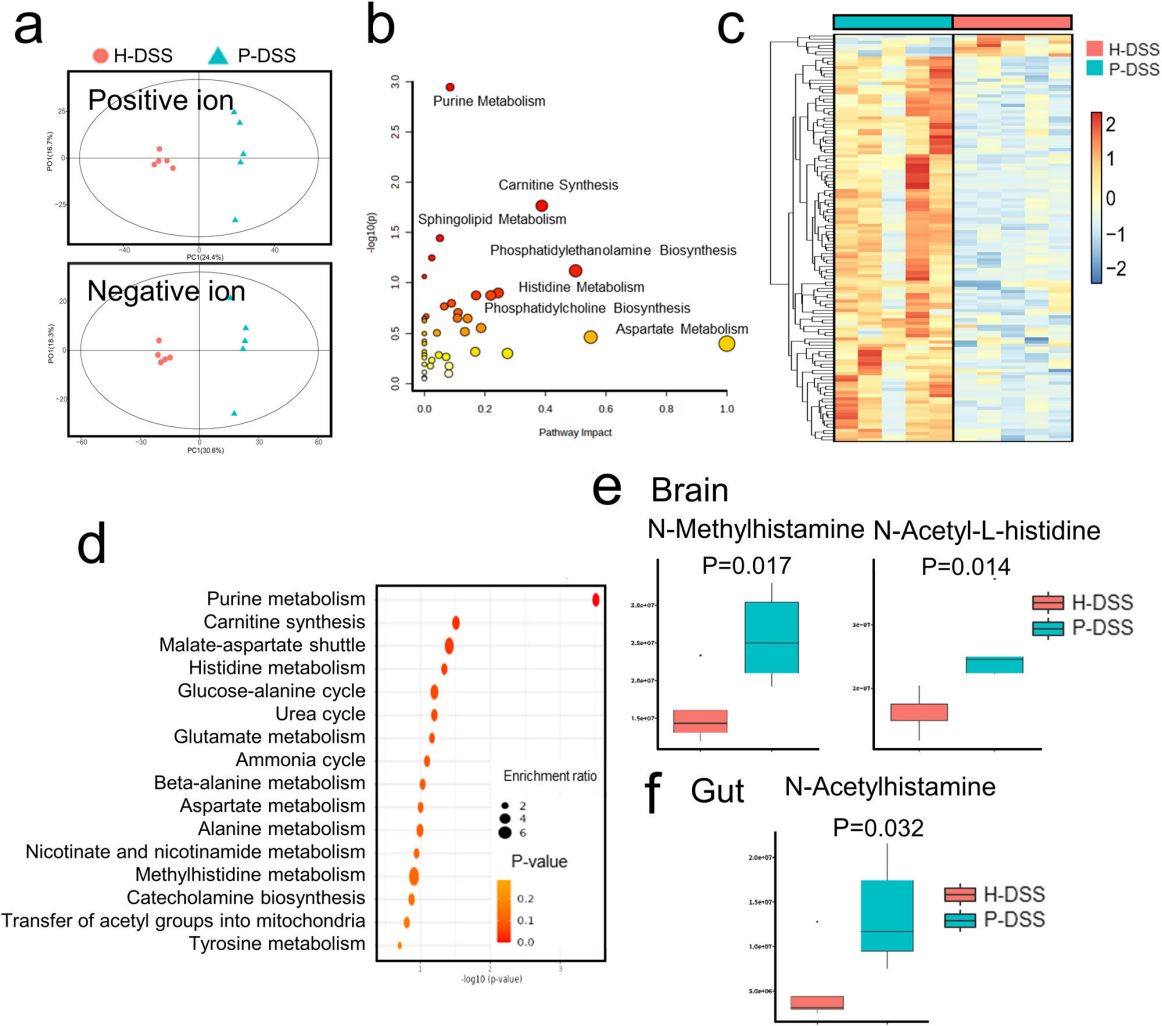
Periodontitis salivary microbiota led to alterations in brain metabolism. **a** OPLS-DA score plots of positive and negative ion modes (*n* = 5 per group). **b** Scatter diagram of metabolite set enrichment analysis. Each plot represents a pathway, plotted by pathway impact on the horizontal axis and log(p) on the vertical axis. **c** Heat map of differential metabolite expression between the P-DSS and H-DSS groups in brain metabolism. **d** Enrichment analysis of brain metabolites in the P-DSS and H-DSS groups. **e** Expression of histamine-related metabolites in the brain, including N-Methylhistamine and N-Acetyl-L-histidine. **f** Expression of N-acetylhistamine in the gut. Statistical analysis was performed by the two-tail *t* test.

Next, we examined brain metabolism to explore key metabolites that affect the anxiety-like behaviors. The results of the OPLS-DA analysis of the positive and negative ion modes showed significant differences in metabolites between the H-DSS and P-DSS groups (Fig. 6a). Enrichment analysis of these metabolites revealed that they were mainly enriched in phosphatidylethanolamine biosynthesis, histidine metabolism, purine metabolism, carnitine synthesis, sphingolipid metabolism, and aspartate metabolism (Fig. 6b). Additionally, pathways related to neurons, such as phosphatidylethanolamine biosynthesis, phosphatidylcholine biosynthesis, and sphingolipid metabolism, were enriched. Differential metabolite expression is shown in Fig. 6c. The major differential metabolites were highly expressed in the P-DSS group; therefore, enrichment analysis of the metabolites elevated in the P-DSS group was performed and showed that purine metabolism, carnitine synthesis, malate-aspartate shuttle, histidine metabolism, and the glucose–alanine cycle were the top five enrichment pathways (Fig. 6d). Histidine metabolites are enriched in both gut contents and the brain. Therefore, we specifically focused on histamine-related metabolites. We examined histamine H1 receptor (H1R) expression in the H-DSS and P-DSS groups; H1R expression was relatively high in the cerebral cortex of the P-DSS group, suggesting that histidine metabolites play a role in the brain (Supplementary Fig. 5c). N-methylhistamine and N-acetyl-L-histidine were highly expressed in the brain metabolism of the P-DSS group (Fig. 6e), whereas NAH was highly expressed in the gut metabolism of the P-DSS group (Fig. 6f). To further explore the relationship between gut microbiota and metabolites, we created a correlation network based on Spearman correlation analysis of brain metabolites, gut metabolites, and gut microbiota (*P* < 0.05). The result showed that *Bacteroidaceae* were closely associated with gut metabolites, including NAH and L-Dopa, whereas only *Enterobacteriaceae* were associated with brain metabolites, suggesting the important role of *Enterobacteriaceae* in the gut-brain axis (Supplementary Fig. 6d; correlations and p-value were shown in Supplementary Table 1). These results suggest that gut microbiota is associated with brain and gut metabolism and that histamine-related metabolites were the key mediators in the effects of PSM on anxiety-like behavior.

### N-acetylhistamine may be a mediator of PSM to promote anxiety through the gut–brain axis

**Fig. 7:**
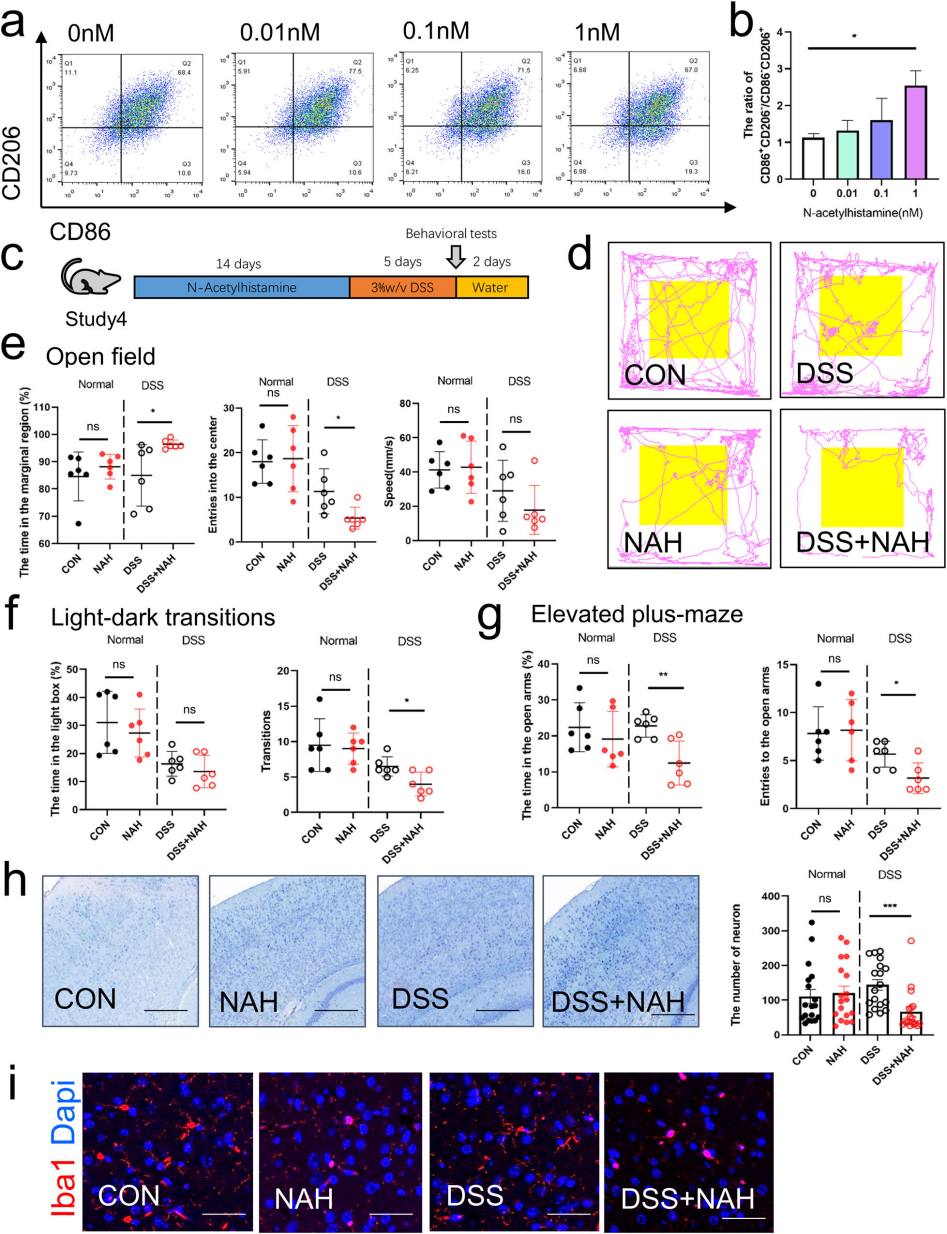
N-acetylhistamine may be a mediator of PSM that promote anxiety through the gut-brain axis. **a** Representative flow cytometry plots of CD86 and CD206 expression in BV-2 cells. **b** Ratio of CD86^+^CD206^–^/CD86^–^CD206^+^ of BV-2 cells at 0,0.01,0.1 and 1 nM concentrations of N-acetylhistamine treatment (*n* = 4). **c** Study design of study 4. A detailed description is provided in the Methods section. **d** Representative image of the open-field track diagram after N-acetylhistamine treatment (*n* = 6 per group). **e** Time spent in the marginal region, frequency of the mice entered the centre zone, and speed in the marginal region in the open field. **f** Time spent in the light box and frequency of light-dark transitions. **g** Time spent in the open arm and the frequency of the mice entered the open arms in the elevated plus-maze. **h** Representative image of Nissl staining and the number of neurons in the N-acetylhistamine treatment experiment, scale bar = 150 μm (For normal neurons counting,6 mice per group, and 3 views of the same site were selected for each mouse to be counted). **i** Representative image of iba1 (red) and DAPI (blue) staining of microglial cells in the N-acetylhistamine treatment experiment, scale bar = 50 μm. Statistical analysis was performed by the one-way ANOVA test with Tukey’s correction (**b**) and two-tailed *t* test (**e**–**h**). Results are shown as mean ± standard error of mean. *p < 0.05, **p < 0.01, ***p < 0. 001.

Finally, to verify the effects of gut metabolites, we first used NAH, the histamine-related substance highly expressed in the gut of the P-DSS group, to stimulate microglia. We found that NAH could increase the expression of CD86^+^/CD206^–^ in mouse BV-2 microglial cells, while the CD206^+^/CD86^–^ expression decreased after the addition of NAH, especially at 1 nM (Fig. 7a, b). Next, we gavaged DSS or non-DSS mice with NAH via 10 nM gavage (Fig. 7c). NAH significantly reduced colon length and increased the histological scores of colitis, indicating that NAH exacerbated colitis in DSS mice, but it did not disrupt the intestinal barrier in the absence of DSS (Supplementary Fig. 7a–e). At behavior level,NAH significantly increased the time spent in the marginal area and decreased the frequency of entering the center zone of the open field in the DSS-treated mice (DSS + NAH group vs. DSS group; Fig. 7d, e). Similarly, the DSS + NAH group had shorter transition times (Fig. 7f) and time spent in the open arms, and a lower frequency of entering the open arms compared with the DSS group (Fig. 7g). At histology level, Nissl staining showed that NAH gavage reduced the number of normal neurons in DSS mice (Fig. 7h). Immunofluorescence revealed NAH-activated microglia, which was most pronounced in the DSS + NAH group (Fig. 7i). However, in non-DSS mice, NAH did not significantly change anxiety-like behaviors, based on the open field, elevated plus-maze, and light–dark transition tests, and the number of normal neurons, suggesting that DSS-induced intestinal disruption was required in NAH-exacerbated anxiety-like behavior. These findings suggest that gut metabolites, such as NAH, are key substances in the promotion of anxiety-like behavior by PSM in DSS mice.

## Discussion

Recently, the role of oral–gut axis has received increasing attention. Periodontitis can affect neuropsychiatric behaviors as well as gut microbiota^19,28,29^; however, the exact mechanism remains to be elucidated. Herein, we discovered that PSM exacerbates DSS-induced anxiety-like behavior via the gut microbiota and its derived metabolites. Histamine-related metabolites may be the main substances that exacerbate anxiety-like behaviors, whereas microglial activation and neuronal reduction may be the relevant mechanisms. Our study suggests that periodontitis affects gut microbiota through saliva, thereby altering anxiety-like behavior and providing evidence for PSM affecting the gut–brain axis.

The oral cavity and intestinal tract are two important bacterial reservoirs that are directly connected. Saliva, a medium shared between these two reservoirs, contains many oral bacteria that are found in the intestinal tract^30,31^. These oral bacteria can cause intestinal barrier function impairment and gut microbiota disorders^19,32^. Thus, periodontitis can affect systemic conditions via the swallowing of bacteria and transmission of inflammatory factors to the body through the bloodstream^7,15^. Oral pathobiont-reactive T-helper 17 cells can be activated by oral bacteria, which migrate to the intestine and worsen colitis^26^. Our results suggest that PSM can exacerbate DSS-induced anxiety-like behavior. Although mixed salivary microbiota reflects the real situation of the host, it is still worthwhile studying how the salivary microbiota influences central nervous system function. Numerous studies have shown that periodontal pathogens, such as *P. gingivalis* and *Fusobacterium nucleatum*, cause central nervous system abnormalities and neuropsychiatric diseases^33–35^. *P. gingivalis* increased the production of IL-1 in thin meningeal cells by inducing synaptic failure^36^. In AD lesions, *P. gingivalis* can also act through capsular polysaccharides and vesicles^37,38^. Yan et al performed silk ligation on the second molar of rats with *Fusobacterium nucleatum ATCC 25586* and found that it contributes to abnormalities in cognitive abilities and brain pathology^39^. Although the mechanisms of these bacteria are not identical, they all cause a range of immune responses in the brain, even the whole body, suggesting that a single periodontitis-pathobiont bacterium can lead to the development of neuropsychiatric disease. Therefore, whether it is particular pathogenic bacteria in saliva, or the combination of mixed microbiota, or increased bacterial levels that play a role in saliva promoting disease development still need to be explored. In addition, as bacteria are naturally present in human saliva, the role of HSM should be considered. Notably, HSM is characterized by the presence of *Streptococcaceae*, and a decrease in *Streptococcus salivarius* leads to increased inflammation in the gut^40^. However, our study revealed that, except for the frequency of transitions in the light–dark transitions, HSM did not affect anxiety-like behaviors when compared with the controls, suggesting that PSM, rather than HSM, is the key factor influencing anxiety-like behaviors. Thus, the role of saliva in healthy individuals remains to be explored.

Previous studies indicate that gut microbiota is a key factor in multiple neuropsychiatric disorders, including emotional, motor, and cognitive functions^41^. Bilateral olfactory bulbectomy mice develop chronic depression and anxiety-like behaviors which are accompanied by alterations in gut microbial characteristics^42^. Germ-free mice exhibit faster gastrointestinal transit, intestinal barrier dysfunction, and anxiety-like behavior after transplantation of feces from patients with irritable bowel syndrome^24^. In contrast, improving the composition of gut microbiota improves depression and anxiety^43^. While antibiotics can alter the composition of gut microbiota, they can remove antibiotic-sensitive gut microbiota. Antibiotics can alter behavior, but this effect returns to baseline within two weeks and these changes are not associated with inflammatory activity, gastrointestinal neurotransmitters, or the vagus nerve, suggesting that antibiotics affect the central nervous system function via the gut microbiota^44^. Our study showed that behavioral differences between the two groups were eliminated after antibiotic treatment, suggesting that antibiotic-sensitive gut microbiota may be a major factor in the PSM exacerbating anxiety-like behaviors. Moreover, probiotics may be beneficial to mental health by improving mood and sleep quality^45^. Therefore, alterations in gut microbiota are directly related to neuropsychiatric disorders. In our study, *Enterobacteriaceae* was the predominant family-level taxa in PSM-gavaged mice and was correlated with brain metabolites. Both Kitamoto et al. and Atarashi et al. showed that *Klebsiella* in *Enterobacteriaceae* is the key bacterium affecting IBD^16,26^. *Enterococcaceae* were more abundant in the feces of patients with IBD and depression than in the feces of patients with IBD but without depression. Gavage of *Enterobacteriaceae*, *Klebsiella*, and *Escherichia coli* alone or in combination caused colitis and depression-like behavior in both germ-free and specific pathogen-free mice^46^. These gram-negative bacteria may alter systemic inflammation and central nervous system function through their by-products, such as lipopolysaccharides and exopolysaccharides. However, the effect of these microbiota on the development of neuropsychiatric diseases requires further investigation.

Gut microbiota-derived metabolites may be an important factor in neuropsychiatric disorders. Gut microbiota can synthesize or consume a wide range of classical neurotransmitters, such as dopamine, serotonin, and γ-aminobutyric acid^47^. Gut-derived metabolites can cross the blood–brain barrier and affect central nervous system activity. Bacteria in the gut can convert tyrosine to 4-ethyl phenyl sulfate (4EPS), which crosses the blood–brain barrier and reduces the maturation of myelin-forming neuro-oligodendrocytes in the brain, leading to anxiety^3^. A clinical trial involving 30 children with autism and gastrointestinal diseases who were administered oral medication AB-2004 had altered metabolic profiles in their blood and urine, including lower 4EPS levels, improved gastrointestinal health, and reduced anxiety^48^. Histamine may also be a key metabolite that influences anxiety and the immune response caused by the dysbiosis of gut microbiota^49,50^. Histamine is involved in multiple physiological processes in the central nervous system through histamine receptors. H1R in neurons and astrocytes can modulate anxiety-like behavior, and inhibition of histamine receptors may be an effective treatment for anxiety^51^. NAH is a marker of histamine metabolism and may be associated with allergic reactions. In our study, NAH also promoted anxiety-like reactions in DSS mice. Previous studies indicate that probiotic *Lactobacillus rhamnosus GG* intervention increases *Bacteroides* count and reduces NAH abundance^52^, suggesting that improvement in gut microbiota reduces the production of such undesirable metabolites. However, histamine can also be produced owing to tissue damage, while the increase in NAH pathogenic bacteria or PSM can also exacerbate colitis; therefore, whether gut microbiota acts by producing undesirable metabolites or exacerbating colitis remains to be elucidated.

Non-neuronal cells in the brain, such as microglia and astrocytes, are critical for providing metabolic support, regulating neurotransmitters, modulating synaptic plasticity, and directly modulating anxiety-like behavior in mice^53,54^. Microglia, the main innate immune cells in the brain, serve as sensors of neuronal activity and microbial-derived molecules^55^. Following infection of central nervous system tissues, microglia can be activated, altering their morphology, phagocytic capacity, and polarization status. In many psychiatric disorders, excessive activation of these cells can lead to overproduction of inflammatory mediators and amplified tissue damage^56^. Selective knockdown of Ucp2 alters reactive oxygen species production, phagocytosis, and the synapse number in microglia, suggesting that the interaction between microglia and neurons is involved in the control of brain function^57^. The alcohol-active kinase Src activates microglia by increasing tumor necrosis factor production, causing anxiety, suggesting that metabolism affects neurons and microglia^22^. Our study showed that PSM and PSM-driven metabolite NAH activate microglia and disrupt cortical neurons. The disruption of sphingolipid metabolism leads to significant neuronal damage and neurodegenerative diseases^58^. Comparably, these neuron-related pathways were enriched in our study, suggesting the possibility of an effect of PSM on neurons. Both the cortex and the hippocampus were involved in anxiety, but we found that pathological changes were more pronounced in cortical regions, probably because histamine and its-related neurons are more closely associated with the cortex^59–61^. However, the hippocampal region has been extensively studied in relation to anxiety and is an anxiety receptor^61^. The role of hippocampal regions in PSM affecting anxiety-like behavior warrant further exploration.

Notably, no significant change was observed in anxiety-like behavior in non-DSS mice, and NAH only worsened anxiety-like behavior in DSS mice, suggesting that intestinal barrier function disruption caused by DSS may be a prerequisite for periodontitis to promote anxiety. However, a limitation of our study was that we observed effects only in the short term, and the results of long-term treatment are unknown. Several studies have also shown that a prolonged influence of periodontitis or PSM can disrupt intestinal barrier function^18,32,62^. In addition to intestinal barrier disruption, periodontitis leads to systemic inflammation^7^, and chronic inflammation is responsible for psychiatric symptoms and nervous system diseases^63^. Prolonged periodontitis may also contribute to neuropsychiatric diseases, including anxiety, trauma, and stress-related disorders^64^. Aragao et al. showed that periodontitis may affect anxiety, although some heterogeneity was present in their systematic review^27^, which may be related to the length of the study and included study types. Hence, it is important to explore the effects of long-term PSM swallowing on the gut.

In conclusion, our results showed that PSM can exacerbate DSS-induced anxiety-like behavior in mice through gut microbiota and metabolic disorders, suggesting that periodontitis salivary microbiota is a factor affecting the gut–brain axis. These findings provide new insights into the important role of periodontitis in gastrointestinal and neuropsychiatric diseases.

## Methods

### Collection and processing of saliva samples

The saliva samples, including 9 patients with periodontitis and 10 healthy individuals, were collected from the Nanjing Stomatological Hospital, Medical School of Nanjing University, which was approved by the Ethics Committee of Nanjing Stomatological Hospital, Medical School of Nanjing University (2019NL-008(KS)). All donors have given informed consent. Approximately 5 mL saliva was collected from each donor. The oral status of the donors is shown in Supplementary Table 2. First, saliva was centrifuged at 200 × *g* for 10 min. The supernatant was collected and suspended in an equal volume (w/v) of phosphate buffered saline (PBS) containing 20% glycerol/PBS, snap-frozen in liquid nitrogen, and stored at −80 °C until use. When required, the desired frozen saliva from different donors were thawed and mixed to ensure that each mouse received the same salivary microbiota each time. And then bacterial mixture was centrifuged at 3300 × *g* for 10 min at 4 °C, suspended in PBS (resuspend each 5 mL saliva in 2 mL PBS), and gavaged into specific-pathogen free (SPF) mice (200 µL per mouse). The inclusion and exclusion criteria were as follows:

#### Inclusion criteria of patients with periodontitis

(1) Patients aged 25–55; (2) 18 retained teeth in the patient’s mouth; and (3) two or more affected teeth of the patient had the following three characteristics: (a) probing depth > 6 mm, (b) clinical attachment loss of 5 mm, and (c) alveolar bone was absorbed more than half of the length of the root.

#### Inclusion criteria of healthy individuals

(1) Patients aged 25–55 years; (2) no teeth lost due to periodontitis; and (3) no significant alveolar bone resorption, loss of attachment, and bleeding on probing.

#### Exclusion criteria

(1) Patients who received periodontal treatment within the last year; (2) long-term use of antibiotics or other drug therapy in the past 6 months; (3) patients with systemic diseases, including obesity, diabetes, immune deficiency, chronic gastrointestinal diseases, cardiovascular and cerebrovascular diseases, and hypertension. (4) other serious oral diseases, such as alveolar abscess; (5) pregnant or lactating patients; and (6) smokers (>5 pieces/day).

### Mice and study design

Eight-week-old wild-type (WT) C57BL/6J male mice were purchased from Beijing Vital River Laboratory and maintained under SPF conditions at Nanjing Agricultural University. All mice were acclimatized for 2 weeks before performing the experiment to avoid fighting. The mice were anaesthetized and euthanized by isoflurane and cervical dislocation at day 21. Each group comprised 6 mice. All animal experiments were approved by the Animal Ethics Committee of Nanjing Agricultural University (PZW2021020). Animal welfare and experimental protocols followed the ARRIVE guidelines (Animal Research: Reporting of In Vivo Experiments). Study 1 involved gavaging the salivary microbiota to the mice to observe the effect of salivary microbiota on anxiety-like behavior. Study 2 involved negating the difference in antibiotic-sensitive gut microbiota between groups by antibiotic treatment to explore the role of gut microbiota in anxiety-like behavior. Study 3 involved transplanting the fecal microbiota altered by salivary microbiota into mice to validate the critical role of gut microbiota in anxiety-like behavior. Study 4 was designed to verify the effects of key gut metabolites on anxiety-like behavior by gavaging N-acetylhistamine. The studies were conducted as follows.

#### Study 1: Treatment with salivary microbiota

A total of 24 mice were randomly assigned to the following groups: (1) control (CON), (2) DSS (before DSS treatment, also assigned to the CON group), (3) HSM gavage (H-DSS), or (4) PSM gavage (P-DSS). Before DSS treatment, the CON and DSS groups (combined in CON and displayed in Supplementary Fig. 2) were gavaged with PBS, and the H-DSS and P-DSS groups were gavaged with HSM and PSM, once every other day for 2 weeks. For H-DSS and P-DSS group, each mouse received the same mixture of salivary microbiota from 10 healthy individuals or 9 patients with periodontitis, respectively. Feces (one pellet of feces per mouse) were collected after the gavage every other day for 2 weeks and processed immediately for fecal microbiota transplantation into Study3 mice at the same day(day 1, 3, 5, …, up to day 13). After 2 weeks of intragastric administration, the CON group received normal water for 5 days, while the DSS, H-DSS and P-DSS groups received 3% DSS (Cat#0216011090; MP Biomedical, USA) for 5 days. All mice underwent behavioral tests on the last day of gavage and DSS exposure. The experimental timeline is shown in Fig. 1f and Supplementary Fig. 2a.

#### Study 2: Antibiotic treatment

A total of 18 mice were randomly assigned to the following groups: (1) antibiotic treatment (ABX), (2) antibiotic treatment via gavage with HSM (H-ABX), or (3) antibiotic treatment via gavage with PSM (P-ABX). Before DSS treatment, the ABX group was gavaged with PBS, and the H-ABX and P-ABX groups were gavaged with HSM or PSM, respectively, once every other day for 2 weeks. All mice were administered a mixture of antibiotics (ampicillin, 1 mg/mL; vancomycin, 0.5 mg/mL; neomycin, 1 mg/mL; metronidazole, 1 mg/mL; Fisher Scientific) in their drinking water during the second week of gavage. The antibiotics were changed every other day. After 2 weeks of intragastric administration, all groups received 3% DSS for 5 days. Behavioral tests were performed on the last day of gavage and DSS exposure. The experimental timeline is shown in Fig. 2a and Supplementary Fig. 3a.

#### Study 3: Fecal microbiota transplantation

A total of 18 mice were randomly assigned to the following groups: (1) transplantation with fecal microbiota from the CON group (FMT), (2) transplantation with fecal microbiota from the HSM- gavage mice (H-FMT), or (3) transplantation with fecal microbiota from the PSM- gavage mice (P-FMT). All feces were collected fresh from Study 1 before DSS treatment on the day of fecal microbiota transplantation (day 1, 3, 5, …, up to day 13). One pellet of feces per mouse was collected and processed immediately for 2 weeks every other day as follows: first, sterile PBS was added and the feces from one group were pooled together and stirred using sterile PBS to create a combined fecal sample of different groups; then, the supernatant was collected after centrifugation at 200 × *g* for 5 min, and diluted to 1.5 mL sterile PBS; finally, 200 µL of the resulting solution was gavaged per mouse. After 2 weeks of gavage, all groups were treated with 3% DSS for 5 days. On the last day of gavage and DSS exposure, behavioral tests were performed. The experimental timeline is shown in Fig. 2f and Supplementary Fig. 3f.

#### Study 4: N-acetylhistamine intervention

A total of 24 mice were randomly assigned to the following groups: (1) CON, (2) DSS, (3) NAH, or (4) DSS + NAH. The CON and DSS groups were gavaged with PBS, and the NAH and DSS + NAH groups were gavaged with 10 nM NAH (CAT#85889; Sigma-Aldrich, USA) per mouse for every other day for 2 weeks. The CON and NAH groups received water for 5 days. The DSS and DSS + NAH groups received 3% DSS for 5 days. All mice underwent a series of behavioral tests on the last day of DSS exposure. The experimental timeline is shown in Fig. 7c.

### Behavioral assays

#### Open field

The open field (40 cm × 40 cm × 40 cm) was composed of white polymethyl methacrylate. Mice were initially placed in the center zone, and their movement was photographed using a camera for 5 min. TopsanLite software (version 2.0) was used to analyze the total distance traveled, time spent in the marginal zone, and entries into the center. More time spent in the marginal region and fewer entries into the center zone indicated increased anxiety-like behaviors. Speed was also examined.

#### Elevated plus-maze

The elevated plus-maze apparatus comprised two open arms (30 cm × 6 cm), two closed arms (30 cm × 6 cm × 15 cm), and a central area. Mice were initially placed in the central zone facing the open arm to explore freely for 3 (Study 1, 2, and 3) or 5 min (Study 4). Mice were considered to enter the open arm when all paws were in the open arm. The time spent in the open arms and number of entries into the open arms were analyzed. Less time spent in the open arms and fewer entries into the open arms indicated increased anxiety-like behaviors.

#### Light–dark transitions

The light–dark box comprised two boxes (25 cm × 25 cm × 25 cm). Mice were initially placed in the light box facing the opening and allowed to explore the entire box freely for 5 min. Mice were considered to enter the light box when all paws were in the light box. The time spent in the light box and total number of side transitions were analyzed. Less time spent in the light box and fewer side transitions indicated increased anxiety-like behaviors.

### Hematoxylin and eosin (HE), periodic acid-Schiff (PAS), and Nissl staining

The colon tissue (from Study 1) and right brain hemispheres samples (from Study 1–4) were collected and fixed in 4% paraformaldehyde for 48 h, embedded in paraffin wax after dehydration, and sectioned at 5 µm. HE staining was performed (CAT#1005, Servicebio, Wuhan, China) to observe the colon morphology. Histological scoring was performed according to methods described in previous studies^16^. PAS was performed according to the manufacturer’s instructions (CAT#G1008, Servicebio) to observe the mucus layer and goblet cells. The right brain hemispheres were stained with toluidine blue (CAT#G1036, Servicebio) to determine the morphology and number of neurons, and the Nissl bodies were stained blue to purple. Three fields of view were selected for each mouse to count the neurons in the cerebral cortex.

### Immunofluorescence

Five-micrometer sections of the right brain hemispheres (from Study 1–4) were incubated with rabbit anti-ionized calcium-binding adapter molecule 1 antibody (Iba1, 1:500; Cat# GB113502, Servicebio) and histamine H1 receptor (H1R,1:500; Cat# 13413-1-ap, Proteintech Group Inc., Wuhan, China) overnight at 4 °C. The sections were then incubated with cyanine 3-conjugated goat anti-rabbit IgG [H + L] secondary antibody (1:500; Cat# GB21303, Servicebio) and HRP goat anti-rabbit secondary antibody (1:500; Cat# GB23204, Servicebio) with FITC-Tyramide (tsa) (Cat# GB1222, Servicebio). Finally, the sections were stained for 10 min in the dark using 4′,6-diamidino-2-phenylindole (tsa) solution (G1012, Servicebio). The images were obtained using a Nikon Eclipse TI-SR fluorescence microscope and the representative images are shown in the paper.

### Cell culture and flow cytometry

BV-2 cells were cultured in Dulbecco’s modified Eagle medium (Cat# C11995500BT; Gbico) and collected for flow cytometry after treatment with NAH for 2 days. The isolated cells were stained with anti-CD86 (1:1000; Cat# 105007, Biolegend, CA, USA) and CD206 (1:1000; Cat# 17-206-82, ebioscience, CA, USA) antibodies per the manufacturer’s instructions. Data were analyzed using Flowjo software (Biosciences, NY, USA).

### 16S rRNA gene sequencing

Each sample (1 ml saliva or 100 mg of caecum content) was collected to extract genomic DNA using the OMEGA Soil DNA Kit (M5635-02) (Omega Bio-Tek, Norcross, GA, USA). After library preparation, sequencing was performed using the NovaSeq-PE250 platform (Illumina Inc., CA, USA). A 16S rRNA gene library was constructed using primers specific for the V3-V4 region, with primers F 5-ACTCCTACGGGAGGCAGCA-3 and R 5-CGGACTACHVGGGTWTCTAAT-3′.Data analyses were performed using the QIIME2 and R packages (V3.3.2). The identities of non-singleton amplicon sequence variations (ASVs) were determined using the Sliva 138. The α-diversity (Shannon, observed species) was estimated by the Wilcoxon test, visualized by the R package “ggplot 2”. β-Diversity was calculated using Bray-Curtis and plotted using principal coordinate analysis (PCoA)^65^. The taxa composition at the phylum and family level was visualized using “qiime taxa barplot” and shown in the paper. Linear discriminant analysis effect size (LefSe) analysis was used to compare between the two groups to identify biomarkers that were statistically different in abundance using the default parameters^66^. Analysis of the composition of microbiomes (ANCOM) was used to identify differentially abundant taxa at family level^67^. Random forest analysis was applied to discriminate the samples from different groups using QIIME2 with default settings^68^. Phylogenetic Investigation of Communities by Reconstruction of Unobserved States (PICRUSt2) was used for prediction of the gut microbiota function^69^.

### qPCR of the bacteria expression at the species level in caecum content

The genomic DNA extracted from caecum content was used as template. DNA concentrations were measured using Nanodrop One. The PowerUp™ SYBR™ Green (CAT# A25742; Thermo Fisher Scientific, USA) was used for qPCR. The primer sequences at the species level were used as following: *Klebsiella* F, 5′-AGCGGAAAAACCGTTAATGCACT-3′; R, 5′-CGATCGGCGCCCATGACTTCAA-3′; *Enterobacter* F, 5′-GAGAACGTCGCCGCCTGGCTGT-3′; R, 5′-AGATACTCTTCCTCCGGCGTTTGCGG-3′.

### Liquid chromatography-mass spectrometry (LC-MS) analysis

Due to the content limitation of sample, we chose 5 mice per group for metabolomics testing. The samples of caecum content and right brain hemispheres (100 mg, ±1%) were weighed and dissolved in 100 μL of acetonitrile/water (1:1, v/v), adequately vortexed, and then centrifuged (13, 400 × *g*, 4 °C, 15 min). Supernatants were collected for LC-MS analysis. Sample separation was performed using UHPLC (1290 Infinity LC, Agilent Technologies). During the experiment, quality control samples were used to evaluate system stability and data reliability. The samples were analyzed in both positive and negative modes of electrospray tandem mass spectrometry. Analyses were performed using UHPLC coupled with quadrupole time-of-flight mass spectrometry (AB SCIEX TripleTOF 6600). Unnecessary information was removed from the spectrum using dynamic exclusion. Raw MS data were converted to MzXML files using ProteoWizard MS Convert, and the XCMS R package was used to detect feature retention time correction and alignment. The metabolites were identified by accuracy mass (<25 ppm), and MS/MS data were matched with online databases, such as HMDB, METLIN, and KEGG. Statistical analyses included the student’s *t*-test and fold change. Only variables with at least one set of non-zero measurements exceeding 50% of the extracted ion features were retained. The experiments were conducted at the Personal Biotechnology Company. Orthogonal partial least-squares discriminant analysis (OPLS-DA) was performed using the R package “muma”. Z-score was standardized to show gut metabolites, using the R command “scale”, and visualized with R package “ggpolt2”. Pathway and enrichment analyses were used by MetaboAnalyst5.0 or R software.

### Statistical analyses

Spearman correlation analyses between bacteria, gut, and brain metabolites (FDR was employed to adjust *p* value, adj *p* < 0.05) were performed using the R software. For normally distributed variables, between-group differences were evaluated using a two-tailed Student’s *t*-test; non-normally distributed variables were analyzed with Wilcoxon tests. For more than three groups, ordinary one-way analysis of variance (ANOVA) with correction of Tukey’s multiple comparison test was used. Significant *P*-values are represented as: *p < 0.05; **p < 0.01, and ***p < 0.001. In addition to the R software, other statistical analyses were performed using Prism 9 (GraphPad Software, San Diego, CA). Data are shown as mean ± standard error of the mean in bar graphs.

### Reporting summary

Further information on research design is available in the Nature Research Reporting Summary linked to this article.

### Supplementary information

Supplementary Information

Reporting Summary

## Data Availability

The microbiome sequencing data have been deposited at the NCBI Sequence Read Archive (SRA) with accession no. PRJNA939965. All raw data will be made available by the corresponding authors upon reasonable request.
