# Supplementary material for: Periodontitis salivary microbiota exacerbates colitis-induced anxiety-like behavior via gut microbiota
Source: NPJ Biofilms Microbiomes. 2023 Dec 7;9:93. doi: 10.1038/s41522-023-00462-9 (PMC10703887; doi:10.1038/s41522-023-00462-9)
Supplement: Supplementary file 1 — Supplementary Information [file 41522_2023_462_MOESM1_ESM.pdf]

Supplementary Table 1. The spearman correlation between *Bacteroidaceae* or *Enterobacteriaceae* and the metabolites of brain and gut

**Gut_*Bacteroidaceae***		
	**Correalation**	**adjust P**
**Gut_N-Acetylhistamine**	0.9758	0.0016
**Gut_L-Dopa**	-0.9030	0.0249
**Gut_D-Phenylalanine**	-0.8667	0.0440
**Gut_N-Acetylputrescine**	0.8667	0.0440
**Gut_Sutterellaceae**	0.8545	0.0516
**Gut_*Enterobacteriaceae***		
	**Correalation**	**adj P**
**Gut_Ergothioneine**	-0.9515	0.0070
**Gut_4-Hydroxystyrene**	-0.9394	0.0104
**Gut_Piperonal**	-0.9394	0.0104
**Gut_L-Glutamine**	-0.9273	0.0151
**Gut_5-Guanidino-3-methyl-2-oxopentanoate**	-0.9273	0.0151
**Gut_Tetrahydrocortisone**	-0.9273	0.0151
**Gut_Cyclopentolate**	-0.9152	0.0197
**Gut_L-Methionine S-oxide**	-0.9152	0.0197
**Gut_allopurinol**	-0.9152	0.0197
**Gut_Anserine**	-0.9030	0.0249
**Gut_ (-)-alpha-Curcumene**	-0.9030	0.0249
**Gut_4-Methoxybenzaldehyde**	-0.9030	0.0249
**Gut_Indole-3-carboxylic acid**	-0.9030	0.0249
**Gut_gamma-L-Glutamyl-D-alanine**	-0.8909	0.0315
**Gut_Beta-Glycerophosphoric acid**	-0.8909	0.0315
**Brain_DL-Lysine**	0.8788	0.0374
**Gut_Styrene Oxide**	-0.8788	0.0374
**Gut_Harmine**	-0.8788	0.0374

**Gut_Nandrolone**	-0.8667	0.0440
**Gut_Aminocyclopropanecarboxylic acid**	-0.8667	0.0440
**Gut_Amino-4-nitrotoluene**	-0.8667	0.0440
**Brain_Indane**	0.8667	0.0440
**Gut_Deoxyguanosine**	-0.8667	0.0440

Supplementary Table 2. The description of donors' oral status

**Number**	**Diagnosis**	**Debris index (DI)**	**Calculus index (CI)**	**Plaque index (PLI)**	**Gingival index (GI)**	**Gender**
**P1**	Periodontitis (stage IV, grade B)	2	2	2	3	Male
**P2**	Periodontitis (stage IV, grade B)	3	3	2	2	Male
**P3**	Periodontitis (stage III, grade B)	2	3	2	2	Male
**P4**	Periodontitis (stage III, grade B)	2	2	2	2	Female
**P5**	Periodontitis (stage IV, grade C)	3	3	3	2	Male
**P6**	Periodontitis (stage IV, grade C)	2	3	2	2	Male
**P7**	Periodontitis (stage IV, grade B)	2	2	2	3	Female
**P8**	Periodontitis (stage IV, grade B)	2	2	2	2	Female
**P9**	Periodontitis (stage IV, grade B)	2	3	2	3	Female
**H1**	Healthy	1	0	1	1	Male

**H2**	Healthy	0	0	0	0	Male
**H3**	Healthy	1	0	0	0	Male
**H4**	Healthy	0	0	0	0	Female
**H5**	Healthy	1	0	1	1	Female
**H6**	Healthy	0	0	0	0	Female
**H7**	Healthy	0	0	0	0	Male
**H8**	Healthy	0	0	0	0	Female
**H9**	Healthy	0	0	0	0	Male
**H10**	Healthy	0	0	0	0	Female

Debris index (DI)

0= no debris or pigment on the tooth surface;1= the debris or pigment covers the tooth surface less than 1/3; 2= the debris or pigment covers the tooth surface 1/3 -2/3; 3= the debris or pigment covers more than 2/3 of the tooth surface.

Calculus index (CI)

0= no calculus; 1= the tooth surface covered by supragingival calculus is less than 1/3; 2= supragingival calculus covering 1/3 - 2/3 of the tooth surface, or there exist subgingival calculus in the tooth neck; 3= subgingival calculus covers more than 2/3 of the tooth surface, or there is thick subgingival calculus in the tooth neck.

Plaque index (PLI)

0= no plaque at gingival margin; 1= thin plaque at gingival margin are not visible, but can be seen by scraping with probe; 2= moderate amount of plaque on gingival margin or adjacent surface; 3= a lot of plaques can be seen in gingival sulcus, gingival margin or adjacent surface.

Gingival index (GI)

0= healthy ;1= gingiva has slight inflammation or mild edema without probing bleeding; 2= moderate inflammation of the gingiva with bright edema and probing bleeding; 3= severe inflammation of the gingiva with swelling gingiva or ulcer and the tendency of spontaneous bleeding.

5

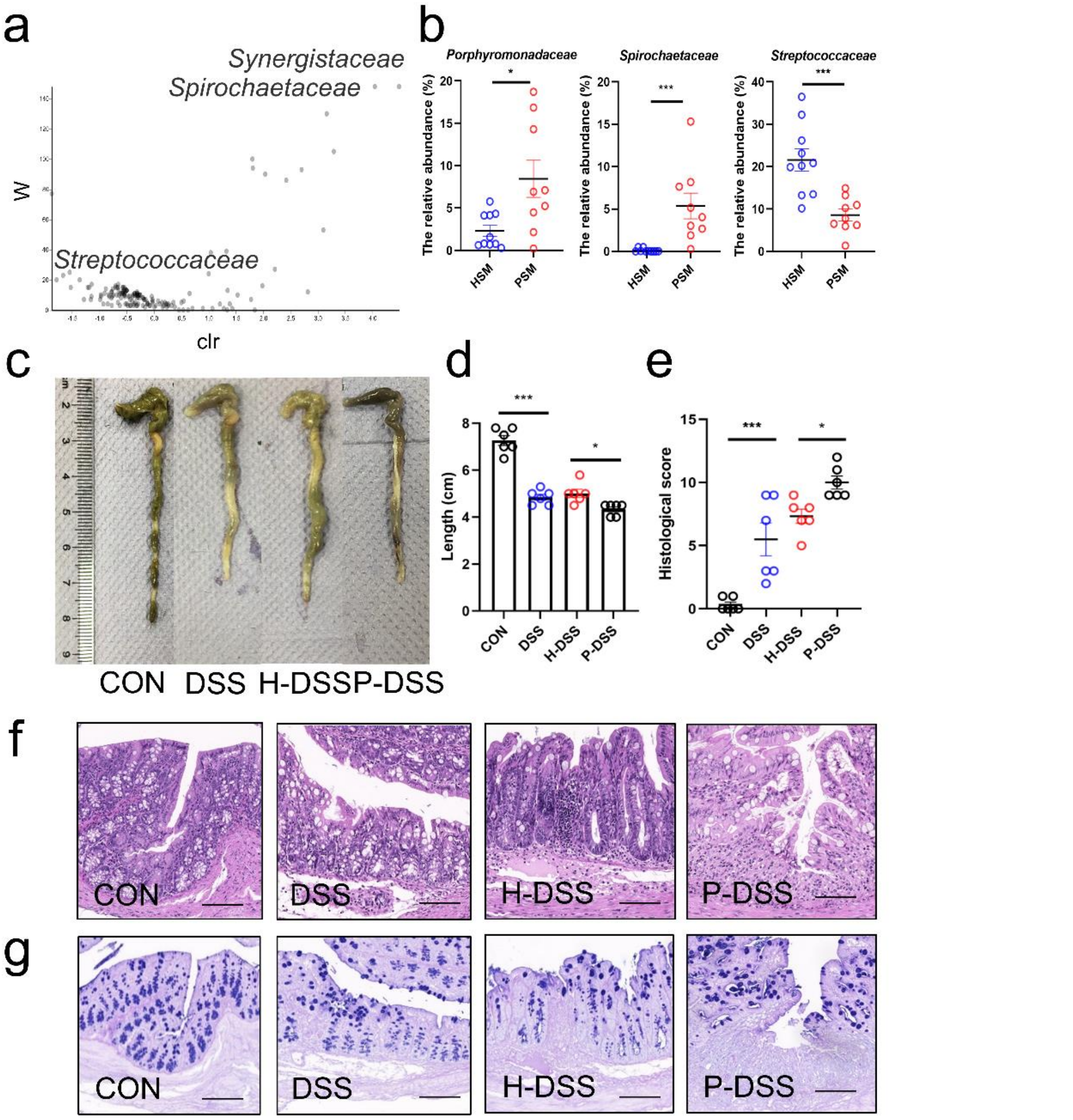

Supplementary Fig. 1 Periodontitis salivary microbiota exacerbates DSS-induced colitis. (a) The ANCOM analysis for salivary microbiota in family level (b) Statistical chart of the relative abundance for key differential microbiota in saliva. (c) Representative image of colon length. (d) Statistical chart of colon length. (e) Histological score. (f) Representative image of haematoxylin and eosin staining, scale bar=100 μm. (g) Representative image of Periodic Acid-Schiff staining, scale bar=100 μm. Statistical analysis was performed by two-tailed t test (b) or the one-way ANOVA test with Tukey's correction (d and e). Results are shown as mean ± standard error of mean. *$p < 0.05$, ***$p < 0.001$.

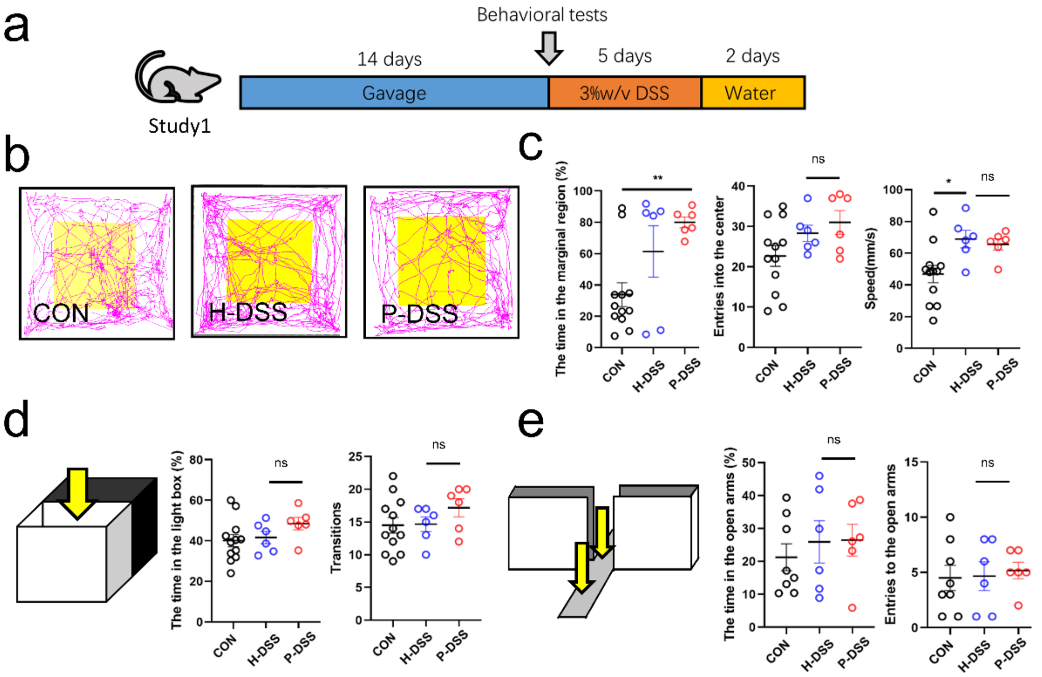

Supplementary Fig. 2 Periodontitis salivary microbiota does not significantly increase anxiety-like behaviour prior to DSS treatment. (a) Schematic representation and study design. Behavioural assays were performed before DSS treatment of mice in study 1. Before DSS treatment, the CON and DSS groups in Study 1 were collectively referred to as CON group (n = 6 per group). (b) Representative image of track diagram of open field. (c) The time spent in the marginal region, the number of times entering the center zone and the speed in the marginal region in the open field. (d) The time spent in the light box and the frequency of transition of light-dark transitions. (e) The time in the open arm and the number of times entering the open arms in the elevated plus-maze. Statistical analysis was performed by the one-way ANOVA test with Tukey's correction. Results are shown as mean ± standard error of mean. $*p < 0.05$, $**p < 0.01$.

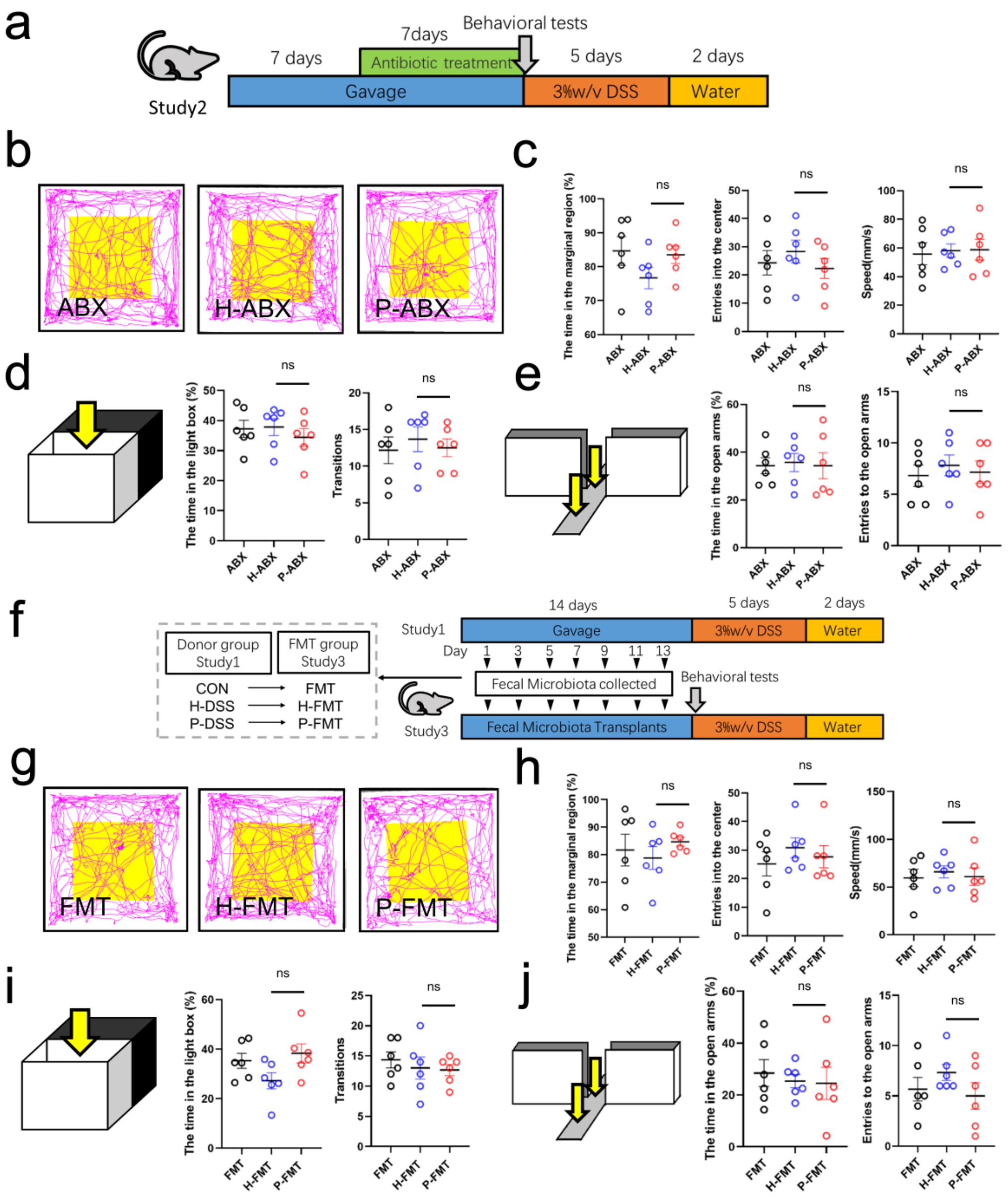

Supplementary Fig. 3 Periodontitis salivary microbiota does not affect anxiety-like behaviour via microbiota before DSS treatment. (a) Study design of antibiotic treatment groups. Behavioural assays were performed before DSS treatment of mice in study 2 (n = 6 per group). (b) Representative image of track diagram of open field after antibiotic treatment. (c)The time spent in the marginal region, the speed in the marginal region and the number of times entering the center zone in the open field. (d) The time spent in the light box and the frequency of transition of light-dark transitions. (e) The time in the open arm and the number of times entering the open arms in the elevated plus-maze. (f) Schematic

representation and study design of faecal microbiota transplantation. The faecal microbiota was from study1 (n = 6 per group). (g) Representative track diagram of open field in the faecal microbiota transplantation. (h) The time spent in the marginal region, the speed in the marginal region and the number of times entering the center zone in the open field. (i) The time spent in the light box and the frequency of transition of light-dark transitions. (j) The time in the open arm and the number of times entering the open arms in the elevated plus-maze. Statistical analysis was performed by two-tailed t test. Results are shown as mean ± standard error of mean.

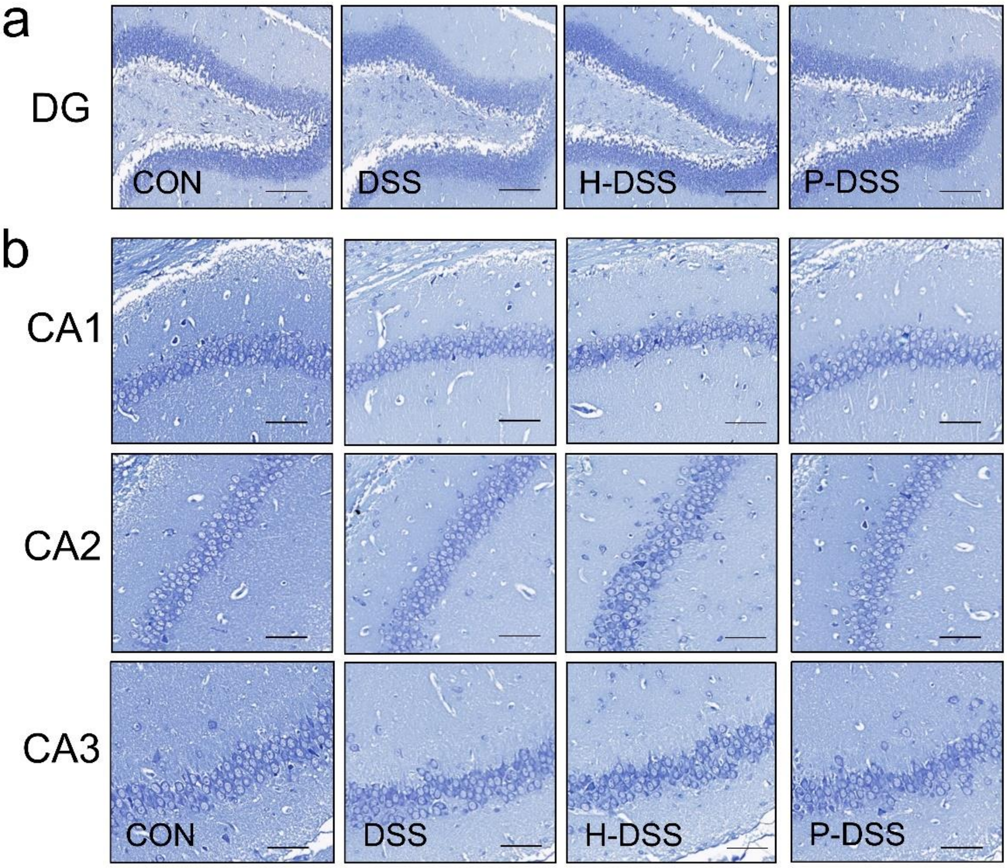

Supplementary Fig. 4 Periodontitis salivary microbiota does not lead to significant changes in neurons in the hippocampal region. (a) Representative image of Nissl staining of the hippocampal DG region of the brain, scale bar=200μm. (b) Representative image of Nissl staining of the hippocampal CA1, CA2 and CA3 region of the brain, scale bar=50μm.

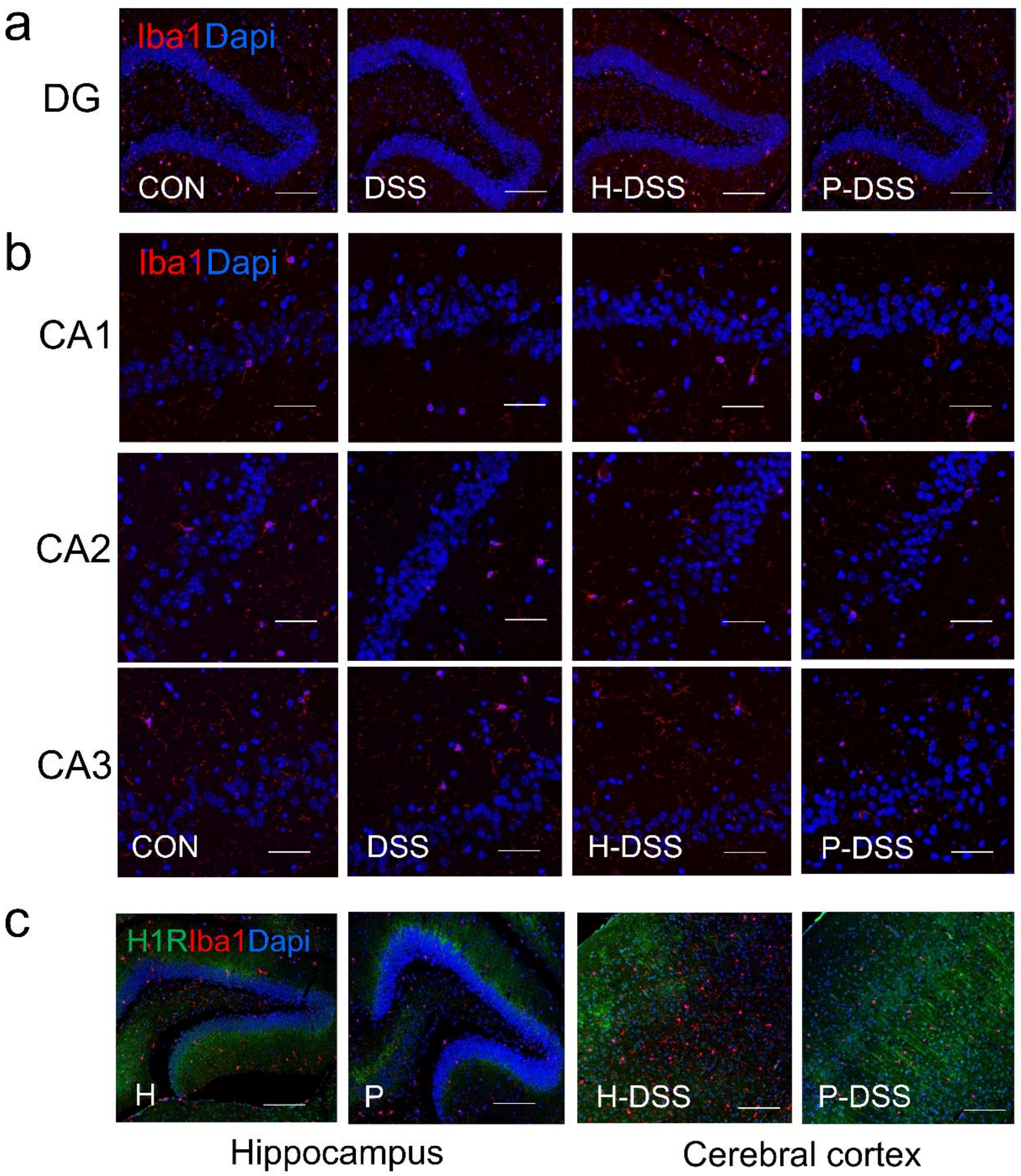

Supplementary Fig. 5 Periodontitis salivary microbiota does not significantly activate microglia in the hippocampal region, but alters cortical histamine H1 receptor. (a) Representative image of Iba1 (red) and Dapi (bule) staining of microglial cell in the hippocampal DG region in the CON, DSS, H-DSS and P-DSS group, scale bar=200μm. (b) Representative image of Iba1 (red) and Dapi (bule) staining of microglial cell in the hippocampal CA1, CA2 and CA3 region in the CON, DSS, H-DSS and P-DSS

group, scale bar=50μm. (c) The expression of histamine H1 receptor (H1R), Iba1 (red) and Dapi (bule) in hippocampus and cerebral cortex of the H-DSS and P-DSS group.

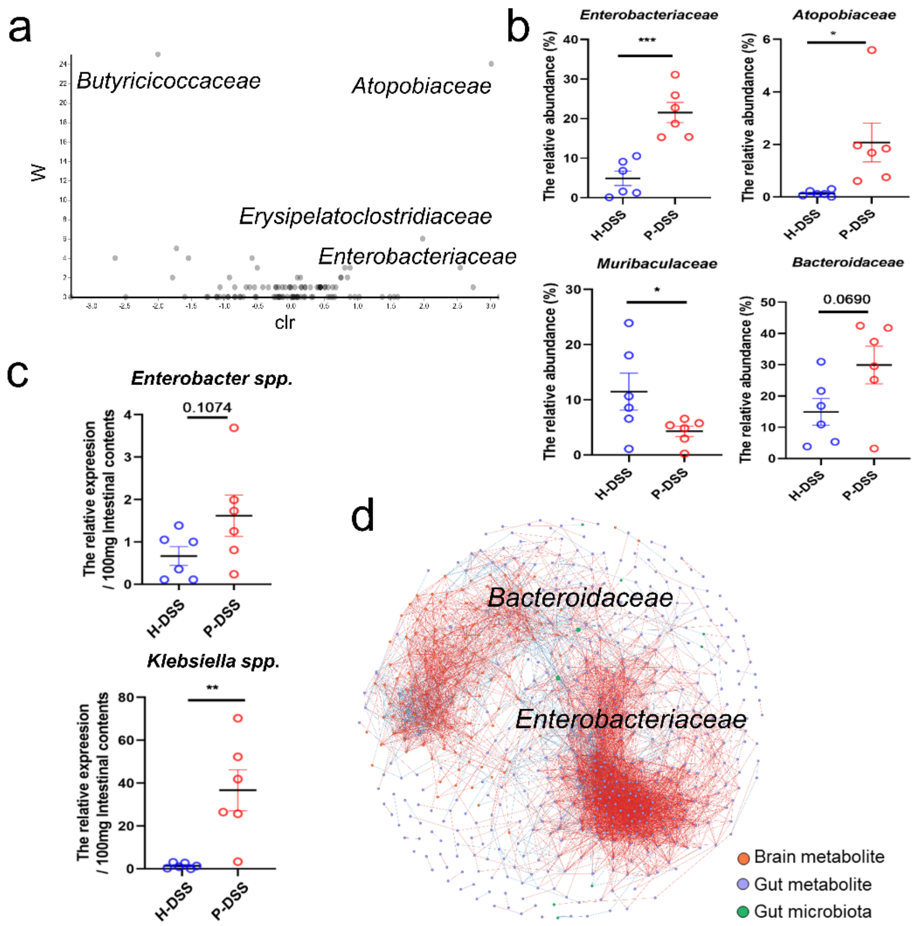

Supplementary Fig. 6 Genus-level detection and multi-omics correlation analysis of gut microbiota. (a) The ANCOM analysis for gut microbiota in family level (b) Statistical chart of the relative abundance for key differential gut microbiota. (c) Species level detection of the dominant bacteria of *Enterobacteriaceae* in P-DSS group by PCR, including *Enterobacter spp.* and *Klebsiella spp*. (d) The spearman correlation network of brain metabolites, gut metabolites, and gut microbiota. Statistical analysis was performed by two-tailed t test. Results are shown as mean ± standard error of mean. *$p < 0.05$, **$p < 0.01$, ***$p < 0.001$.

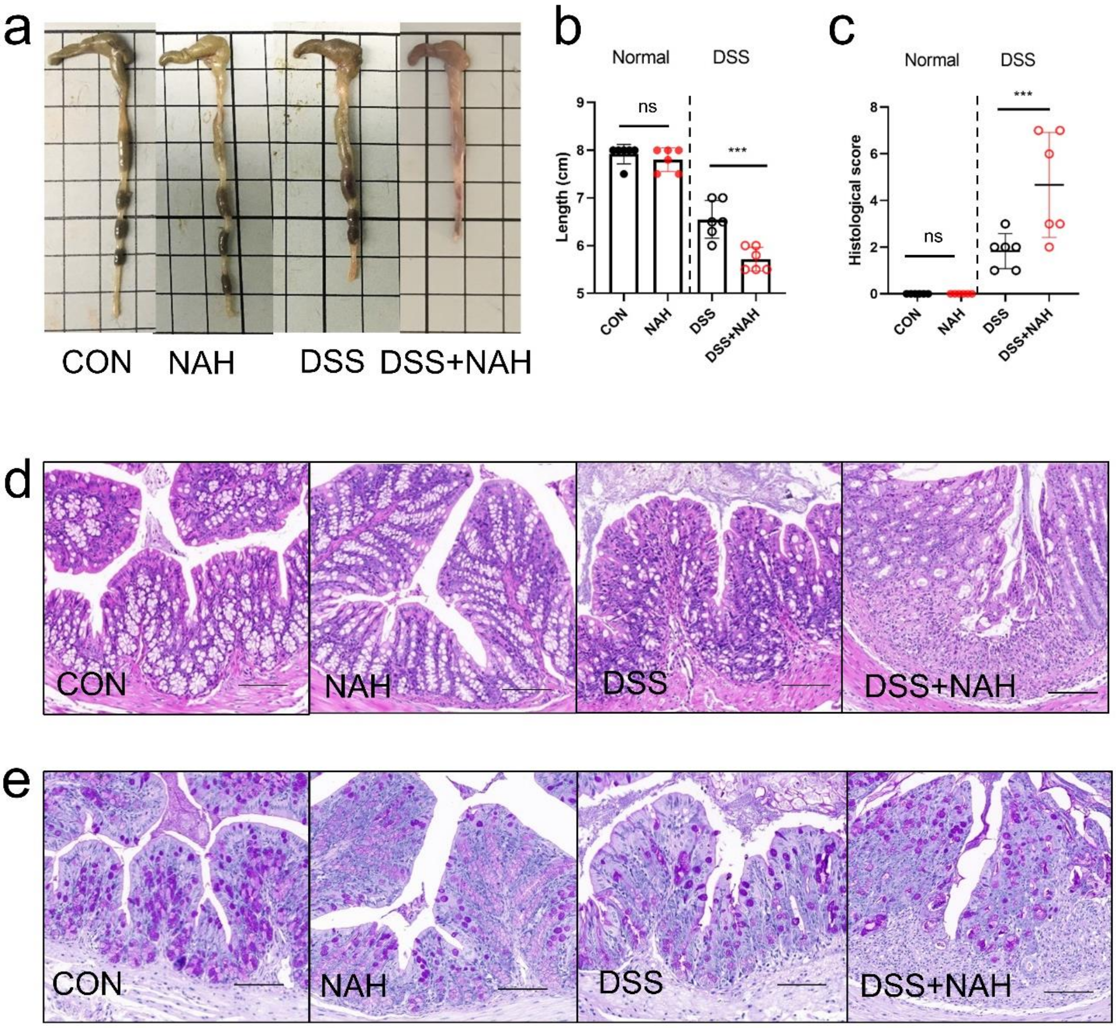

Supplementary Fig. 7 N-acetylhistamine exacerbated DSS-induced colitis. (a) Representative image of colon length. (b) Statistical chart of colon length. (c) Histological score. (d) Representative image of haematoxylin and eosin staining, scale bar=100 μm. (e) Representative image of Periodic Acid-Schiff staining, scale bar=100 μm. Statistical analysis was performed by two-tailed t test. Results are shown as mean ± standard error of mean. ***p <0.001.
