# Supplementary material for: Periodontitis salivary microbiota exacerbates colitis-induced anxiety-like behavior via gut microbiota
Source: NPJ Biofilms Microbiomes. 2023 Dec 7;9:93. doi: 10.1038/s41522-023-00462-9 (PMC10703887; doi:10.1038/s41522-023-00462-9)
Supplement: Supplementary file 2 — Reporting Summary [file 41522_2023_462_MOESM2_ESM.pdf]

nature portfolio

Corresponding author(s): Yanfen Li and Fuhua Yan:

Last updated by author(s): Nov 11, 2023

Reporting Summary

Nature Portfolio wishes to improve the reproducibility of the work that we publish. This form provides structure for consistency and transparency in reporting. For further information on Nature Portfolio policies, see our Editorial Policies and the Editorial Policy Checklist.

Statistics

For all statistical analyses, confirm that the following items are present in the figure legend, table legend, main text, or Methods section.

n/a	Confirmed	
☐	☒	The exact sample size (*n*) for each experimental group/condition, given as a discrete number and unit of measurement
☐	☒	A statement on whether measurements were taken from distinct samples or whether the same sample was measured repeatedly
☐	☒	The statistical test(s) used AND whether they are one- or two-sided<br>*Only common tests should be described solely by name; describe more complex techniques in the Methods section.*
☒	☐	A description of all covariates tested
☐	☒	A description of any assumptions or corrections, such as tests of normality and adjustment for multiple comparisons
☐	☒	A full description of the statistical parameters including central tendency (e.g. means) or other basic estimates (e.g. regression coefficient) AND variation (e.g. standard deviation) or associated estimates of uncertainty (e.g. confidence intervals)
☐	☒	For null hypothesis testing, the test statistic (e.g. *F*, *t*, *r*) with confidence intervals, effect sizes, degrees of freedom and *P* value noted<br>*Give P values as exact values whenever suitable.*
☒	☐	For Bayesian analysis, information on the choice of priors and Markov chain Monte Carlo settings
☒	☐	For hierarchical and complex designs, identification of the appropriate level for tests and full reporting of outcomes
☐	☒	Estimates of effect sizes (e.g. Cohen's *d*, Pearson's *r*), indicating how they were calculated

Our web collection on statistics for biologists contains articles on many of the points above.

Software and code

Policy information about availability of computer code

Data collection	QIIME2 and R packages (V3.3.2) for 16S rRNA gene sequencing; Prism 9 for statistical analyses
Data analysis	16S rRNA gene sequencing: The identities of non-singleton amplicon sequence variations (ASVs) were determined using the Sliva 138. The α-diversity (Shannon, observed species) was estimated by the Wilcoxon test, visualized by the R package “ggplot 2”. β-Diversity was calculated using Bray-Curtis and plotted using principal coordinate analysis (PCoA)63. The taxa composition at the phylum and family level was visualized using “qiime taxa barplot” and shown in the paper. Linear discriminant analysis effect size (LefSe) analysis was used to compare between the two groups to identify biomarkers that were statistically different in abundance using the default parameters64. Analysis of the composition of microbiomes (ANCOM) was used to identify differentially abundant taxa at family level 65. Random forest analysis was applied to discriminate the samples from different groups using QIIME2 with default settings.<br>Statistical analyses : Spearman correlation analyses between bacteria, gut, and brain metabolites (FDR was employed to adjust p value, adj p<0.05) were performed using the R software. For normally distributed variables, between-group differences were evaluated using a two-tailed Student’s t-test; non-normally distributed variables were analyzed with Wilcoxon tests. For more than three groups, ordinary one-way analysis of variance (ANOVA) with correction of Tukey’s multiple comparison test was used.

For manuscripts utilizing custom algorithms or software that are central to the research but not yet described in published literature, software must be made available to editors and reviewers. We strongly encourage code deposition in a community repository (e.g. GitHub). See the Nature Portfolio guidelines for submitting code & software for further information.

Data

Policy information about availability of data

All manuscripts must include a data availability statement. This statement should provide the following information, where applicable:

- Accession codes, unique identifiers, or web links for publicly available datasets
- A description of any restrictions on data availability
- For clinical datasets or third party data, please ensure that the statement adheres to our policy

The microbiome sequencing data have been deposited at the NCBI Sequence Read Archive (SRA) with accession no. PRJNA939965. All raw data will be made available by the corresponding authors upon reasonable request.

Research involving human participants, their data, or biological material

Policy information about studies with human participants or human data. See also policy information about sex, gender (identity/presentation), and sexual orientation and race, ethnicity and racism.

Reporting on sex and gender	9 patients with periodontitis (4 female and 5 male)and 10 healthy individuals(5 male and 5female)
Reporting on race, ethnicity, or other socially relevant groupings	ethnicity:china, han
Population characteristics	9 patients with periodontitis and 10 healthy individuals
Recruitment	First, saliva was centrifuged at 1,000rpm for 10 min. The supernatant was collected and suspended in an equal volume (w/v) of phosphate buffered saline (PBS) containing 20% glycerol/PBS, snap-frozen in liquid nitrogen, and stored at -80°C until use. When required, the desired frozen saliva from different donors were thawed and mixed to ensure that each mouse received the same salivary microbiota each time. And then bacterial mixture was centrifuged at 3,300× g for 10 min at 4°C, suspended in PBS (resuspend each 5 mL saliva in 2 mL PBS), and gavaged into specific-pathogen free (SPF) mice (200 µL per mouse).
Ethics oversight	the Ethics Committee of Nanjing Stomatological Hospital, Medical School of Nanjing University

Note that full information on the approval of the study protocol must also be provided in the manuscript.

Field-specific reporting

Please select the one below that is the best fit for your research. If you are not sure, read the appropriate sections before making your selection.

☒ Life sciences ☐ Behavioural & social sciences ☐ Ecological, evolutionary & environmental sciences

For a reference copy of the document with all sections, see nature.com/documents/nr-reporting-summary-flat.pdf

Life sciences study design

All studies must disclose on these points even when the disclosure is negative.

Sample size	84 mice was used in the study.
Data exclusions	Due to the content limitation of sample, we chose 5 mice per group for metabolomics testing.
Replication	Each group comprised 6 mice.
Randomization	All the mice were randomly assigned to each group according to simple random sampling.
Blinding	J.Q.and J.L. were aware of the group allocation during the allocation. The following experiment management is carried out by S.C., X.Z., Q.T. and the lab manager.

Reporting for specific materials, systems and methods

We require information from authors about some types of materials, experimental systems and methods used in many studies. Here, indicate whether each material, system or method listed is relevant to your study. If you are not sure if a list item applies to your research, read the appropriate section before selecting a response.

Materials & experimental systems

n/a	Involved in the study
☐	☒ Antibodies
☒	☐ Eukaryotic cell lines
☒	☐ Palaeontology and archaeology
☐	☒ Animals and other organisms
☒	☐ Clinical data
☒	☐ Dual use research of concern
☒	☐ Plants

Methods

n/a	Involved in the study
☒	☐ ChIP-seq
☐	☒ Flow cytometry
☒	☐ MRI-based neuroimaging

Antibodies

Antibodies used	rabbit anti-ionized calcium-binding adapter molecule 1 antibody (Iba1, Cat# GB113502; Servicebio) , histamine H1 receptor (H1R; Cat# 13413-1-ap, Proteintech Group Inc., Wuhan, China); anti-CD86 (Cat# 105007, Biolegend, CA, USA), CD206 (Cat# 17-2061-82, ebioscience, CA, USA) antibodies
Validation	CD86:https://www.biolegend.com/en-us/products/pe-anti-mouse-cd86-antibody-256<br>CD206:https://www.thermofisher.cn/cn/zh/antibody/product/CD206-MMR-Antibody-clone-MR6F3-Monoclonal/17-2061-82<br>iba1:https://www.servicebio.cn/goodsdetail?id=6764<br>H1R:https://www.ptgcn.com/products/HRH1-Antibody-13413-1-AP.htm

Animals and other research organisms

Policy information about studies involving animals; ARRIVE guidelines recommended for reporting animal research, and Sex and Gender in Research

Laboratory animals	C57BL/6J from Beijing Vital River Laboratory
Wild animals	Eight-week-old wild-type
Reporting on sex	male
Field-collected samples	All mice were acclimatized for 2 weeks before performing the experiment. The mice was maintained under SPF conditions at Nanjing Agricultural University
Ethics oversight	Animal Ethics Committee of Nanjing Agricultural University

Note that full information on the approval of the study protocol must also be provided in the manuscript.

Flow Cytometry

Plots

Confirm that:

- ☒ The axis labels state the marker and fluorochrome used (e.g. CD4-FITC).
- ☒ The axis scales are clearly visible. Include numbers along axes only for bottom left plot of group (a 'group' is an analysis of identical markers).
- ☒ All plots are contour plots with outliers or pseudocolor plots.
- ☒ A numerical value for number of cells or percentage (with statistics) is provided.

Methodology

Sample preparation	BV-2 cells were cultured in Dulbecco’s modified Eagle medium (Ref# C11995500BT; Gbico) and collected for flow cytometry after treatment with NAH for 2 days. The isolated cells were stained with anti-CD86 (Cat# 105007, Biolegend, CA, USA) and CD206 (Ref# 17-2061-82, ebioscience, CA, USA) antibodies per the manufacturer’s instructions.
Instrument	FACSCablibur
Software	Flowjo software (Biosciences, NY, USA).
Cell population abundance	BV-2 cells

Gating strategy	Gates were drawn at approximately 1.5*10^101 for CD86 and CD206, with values greater than that being positive and values less than that being negative

☒ Tick this box to confirm that a figure exemplifying the gating strategy is provided in the Supplementary Information.
